# The covariance environment defines cellular niches for spatial inference

**DOI:** 10.1038/s41587-024-02193-4

**Published:** 2024-04-02

**Authors:** Doron Haviv, Ján Remšík, Mohamed Gatie, Catherine Snopkowski, Meril Takizawa, Nathan Pereira, John Bashkin, Stevan Jovanovich, Tal Nawy, Ronan Chaligne, Adrienne Boire, Anna-Katerina Hadjantonakis, Dana Pe’er

**Affiliations:** 1 Computational and Systems Biology Program, Sloan Kettering Institute, Memorial Sloan Kettering Cancer Center, New York, NY USA; 2 Tri-Institutional Training Program in Computational Biology and Medicine, Weill Cornell Medicine, New York, NY USA; 3 Human Oncology & Pathogenesis Program, Memorial Sloan Kettering Cancer Center, New York, NY USA; 4 Developmental Biology Program, Sloan Kettering Institute, Memorial Sloan Kettering Cancer Center, New York, NY USA; 5 S2 Genomics, Livermore, CA USA; 6 Department of Neurology, Memorial Sloan Kettering Cancer Center, New York, NY USA; 7 Brain Tumor Center, Memorial Sloan Kettering Cancer Center, New York, NY USA; 8 Howard Hughes Medical Institute, New York, NY USA

**Keywords:** Computational models

## Abstract

A key challenge of analyzing data from high-resolution spatial profiling technologies is to suitably represent the features of cellular neighborhoods or niches. Here we introduce the covariance environment (COVET), a representation that leverages the gene–gene covariate structure across cells in the niche to capture the multivariate nature of cellular interactions within it. We define a principled optimal transport-based distance metric between COVET niches that scales to millions of cells. Using COVET to encode spatial context, we developed environmental variational inference (ENVI), a conditional variational autoencoder that jointly embeds spatial and single-cell RNA sequencing data into a latent space. ENVI includes two decoders: one to impute gene expression across the spatial modality and a second to project spatial information onto single-cell data. ENVI can confer spatial context to genomics data from single dissociated cells and outperforms alternatives for imputing gene expression on diverse spatial datasets.

## Abstract (web summary)

Using gene–gene covariance structure to represent cellular neighborhoods improves the integration of single-cell RNA sequencing and spatial transcriptomics data.

## Main

Intense interest in cellular interactions and tissue context has spurred the growth of multiplexed spatial transcriptomics and antibody-based technologies, sparking the need for computational approaches to identify biological patterns within tissues^1–5^. The local neighborhood, or niche, of a cell is a useful resolution for defining cell interactions; it may represent functional anatomical subunits (such as stem cell niches) and is a basis for identifying larger spatial patterns. However, efficient representations of the cellular microenvironment that retain the full richness of the data and can be used to effectively compare niches are lacking^6^. At the same time, there is a need to address the limited molecular plexity of high-resolution spatial profiling technologies^7^.

Most methods for analyzing spatial data characterize each niche by tabulating discrete cell types within a given region^8–11^. Although these have generated important discoveries^2,8^, they were developed for low-plex antibody-based imaging methods that devote most markers to cell typing. Spatial transcriptomics methods, including commercial platforms, can now profile hundreds of genes^12–18^, meaning that analysis at the cell type level leads to substantial information loss. In single-cell genomics, the switch from discrete cell typing to continuous approaches, such as diffusion maps^19^ and pseudotime^20,21^, has driven remarkable discovery. Moreover, setting thresholds for continuous cellular phenotypes is subjective and invokes problems of instability and bias. Even within highly discrete cell types, vast and meaningful variation often exists, such as the spectrum of activated and metabolic states within immune cell types^22–24^.

Thus, a niche representation is needed that considers the full measured expression and its continuous nature and that enables robust, efficient comparisons. We propose a representation that goes beyond cell typing and preserves complex patterns of gene expression, including covariation in genes across cell states. Specifically, we developed the covariance environment (COVET), a compact representation of a cell’s niche that assumes that interactions between the cell and its environment create biologically meaningful covariate structure in gene expression between cells of the niche. We developed a corresponding distance metric that unlocks the ability to compare and analyze niches using the full toolkit of approaches currently employed for cellular phenotypes, including dimensionality reduction, spatial gradient analysis and clustering.

Imaging-based spatial transcriptomics technologies face issues that practically limit quantification to hundreds of genes. Some methods can impute spatial information for genes not measured in the spatial modality, by integrating matched single-cell RNA sequencing (scRNA-seq) data^9,25,26^. However, integration methods do not explicitly model cellular microenvironment context from the spatial data, thereby limiting inference power.

To achieve transcriptome-wide spatial inference, we developed environmental variational inference (ENVI), a conditional variational autoencoder (CVAE)^27,28^, that simultaneously incorporates scRNA-seq and spatial data into a single embedding. ENVI leverages the covariate structure of COVET as a representation of cell microenvironment and achieves total integration by encoding both genome-wide expression and spatial context (the ability to reconstruct COVET matrices) into its latent embedding. Our approach is effective on data from a variety of multiplexed spatial technologies and outperforms other methods in accurately imputing the expression of genes in diverse developmental contexts. ENVI can also be used to project valuable spatial information onto dissociated scRNA-seq data and can capture continuous variation along spatial axes across large complex tissue regions.

## Results

### COVET defines spatial neighborhoods

To move beyond cell type fraction and to characterize niches in a manner that leverages measured genes and enables quantitative comparison, we developed the COVET framework. Our core assumption is that a cell affects—and is also affected by—cells in its vicinity, generating covarying patterns of expression among the interacting cells. Our framework includes three components: (1) COVET, a robust per-cell representation of neighborhood information based on a modified formulation of gene–gene covariance among niche cells; (2) a distance metric that is essential for comparing and interpreting niches; and (3) an algorithm to efficiently compute this distance metric. Unlike mean expression, gene–gene covariance captures the relationships among genes and cell states that are shaped by cellular interplay within the niche. These relationships are rich, stable and enriched for biological signal; moreover, they contain substantial hidden information from unmeasured genes, providing an advantage for imputation tasks.

**Fig. 1:**
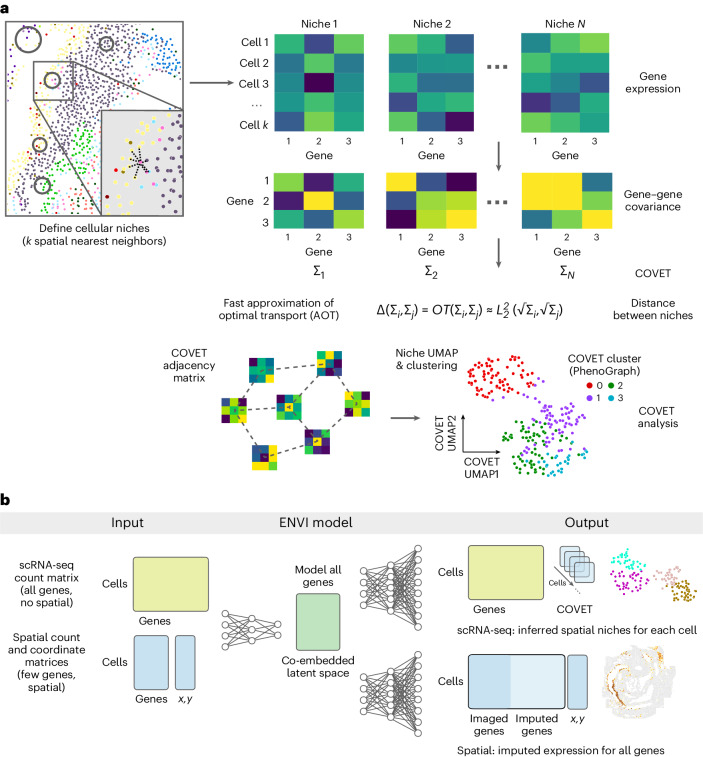
A covariance-based framework characterizes spatial niches and powers single-cell and spatial data integration for robust transcriptome-wide spatial inference. **a**, Schematics indicate steps in spatial covariance computation and ENVI operation. Each COVET matrix characterizes a cell’s niche, comprising *k* nearest spatial neighbors, based on the shifted covariance of gene expression within the niche. Shifted covariance is calculated relative to mean expression in the sample, enabling meaningful comparison of niches. Distance between niches is determined by an efficient approximation of optimal transport. The COVET adjacency matrix, based on AOT as the spatial similarity metric, can be used directly for other downstream spatial analyses, such as dimensionality reduction and clustering, where cells are grouped together by similar environment rather than by expression. **b**, ENVI is a conditional autoencoder that simultaneously embeds scRNA-seq and multiplexed spatial transcriptomic data into a unified latent embedding. ENVI models all genes (including those not imaged with spatial transcriptomics) and uses the COVET framework to represent information about cellular environment. An environment decoder allows ENVI to project spatial information onto single-cell data, and an expression decoder, which also includes genes captured only in the single-cell data, enables imputation of spatial expression transcriptome wide.

To calculate COVET, we first define the niche of each cell in a dataset by the *k* spatial nearest neighbors of that cell and then compute each niche’s gene–gene shifted covariance matrix (Fig. 1a and Methods). Shifted covariance modifies the classic covariance formulation by using mean expression across the entire dataset rather than local mean expression as a reference. This constructs each cell’s covariance matrix relative to the entire population and critically enables direct comparison between niches, highlighting their shared and unique features. Gene–gene covariance provides the additional benefit of being more robust to technical artifacts^23^, facilitating integration across technologies.

**Extended Data Fig. 1:**
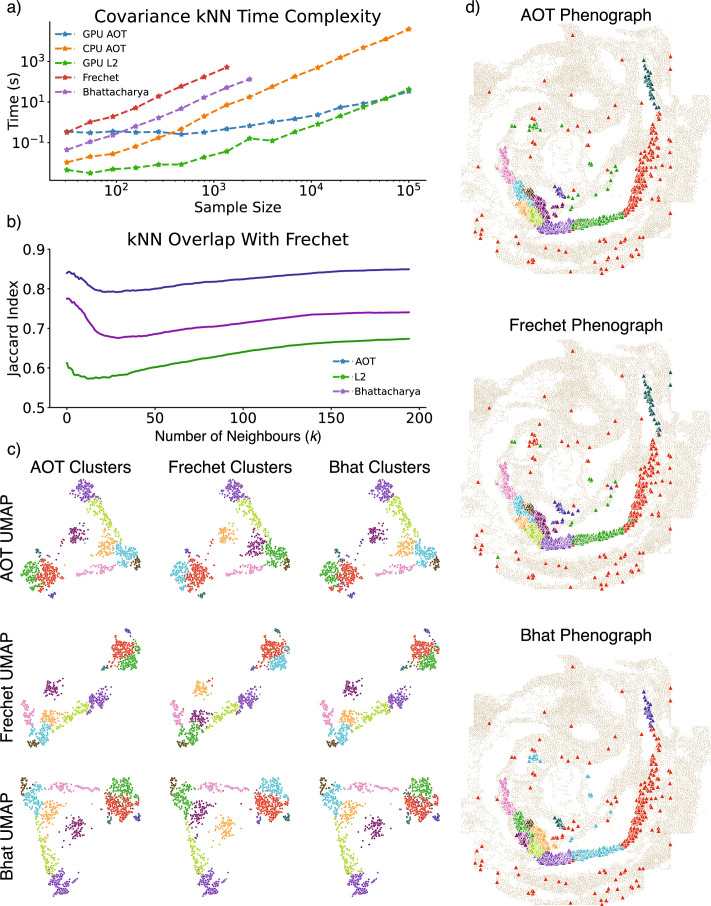
Approximate optimal transport (AOT) yields similar results to optimal transport and Bhattacharyya distance, but more efficiently. **a**, Run times for computing the kNN graph between sets of randomly generated covariance matrices at various sample sizes. Both axes are in log scale. Fréchet and Bhattacharyya run times are not shown for samples larger than 4,000 cells due to out-of-memory error on a 768-GB, 64-core computing cluster. **b**, Agreement between the true Fréchet and AOT, Bhattacharyya and standard L2 kNN graphs, expressed as Jaccard Index values and computed on COVET matrices of pharyngeal mesoderm cells in the seqFISH embryogenesis dataset. **c**, COVET UMAP embeddings and PhenoGraph clustering of seqFISH pharyngeal mesoderm by different metrics, colored by PhenoGraph clusters of each. **d**, seqFISH data from pharyngeal mesoderm, colored by PhenoGraph clustering of COVET matrices according to each distance metric. Bhat, Bhattacharyya.

Despite being a compact and powerful representation of the niche, COVET requires a metric for comparison. Niche similarity cannot be determined by simply subtracting the cell-by-gene expression of matrices of two niches, because the result depends on cell order, which is set arbitrarily (it will change if an image is rotated, for example). We, thus, seek to quantify niche similarity in a permutation-invariant manner, for which the Fréchet distance provides a closed-form solution^29^. However, calculating Fréchet distance is computationally intractable, so we developed an approximation (approximate optimal transport (AOT)) that reduces runtime by over an order of magnitude and is substantially faster than another common metric, the Bhattacharyya distance^30^ (Extended Data Fig. 1a). AOT yields similar results to true optimal transport, and a GPU implementation takes under 1 min to compute the cell–cell AOT distance matrix of 100,000 cells (Extended Data Fig. 1b–d).

As AOT can be computed via Euclidean distance, which underlies many standard single-cell analyses, such as clustering^31^, diffusion components^32^ and uniform manifold approximation and projection (UMAP)^33^, niches can now be analyzed with the same algorithms designed to analyze phenotypes. Clustering niches can characterize canonical environments; visualization can be used to observe their relationships; and trajectory analysis can capture continuous trends, enabling facile interpretation. COVET thus provides a rich, robust and computationally efficient representation of cellular niches, derived from a mathematically principled formulation based on optimal transport.

### The ENVI algorithm

ENVI employs a conditional variational autoencoder to infer spatial context in scRNA-seq data and impute missing genes in spatial data, by mapping both modalities to a common embedding (Fig. 1b and Methods). Unlike other CVAEs used for spatial inference^25,34,35^, which only model genes measured in both modalities, ENVI explicitly models spatial information and gene expression genome wide. More importantly, it uses the COVET matrix to represent spatial information and simultaneously trains on samples from both spatial and single-cell datasets, optimizing a single latent space to decode the full transcriptome and spatial context for both modalities.

ENVI architecture includes a single encoder for both spatial and single-cell genomics data and two decoder networks: one for the full transcriptome and the second for the COVET matrix, providing spatial context. The requisite for decoding the spatial niche (and the use of a second decoder) is a unique aspect of ENVI. Intuitively, ENVI uses gene expression in the cell paired with its niche information (COVET) to learn an ‘environment’ regression model, which infers spatial context from gene expression input, and, simultaneously, an ‘imputation’ regression model trained to reproduce the full scRNA-seq dataset from the gene subset profiled by spatial transcriptomics. The nonlinear network architecture can capture complex dependencies between the variables.

**Extended Data Fig. 2:**
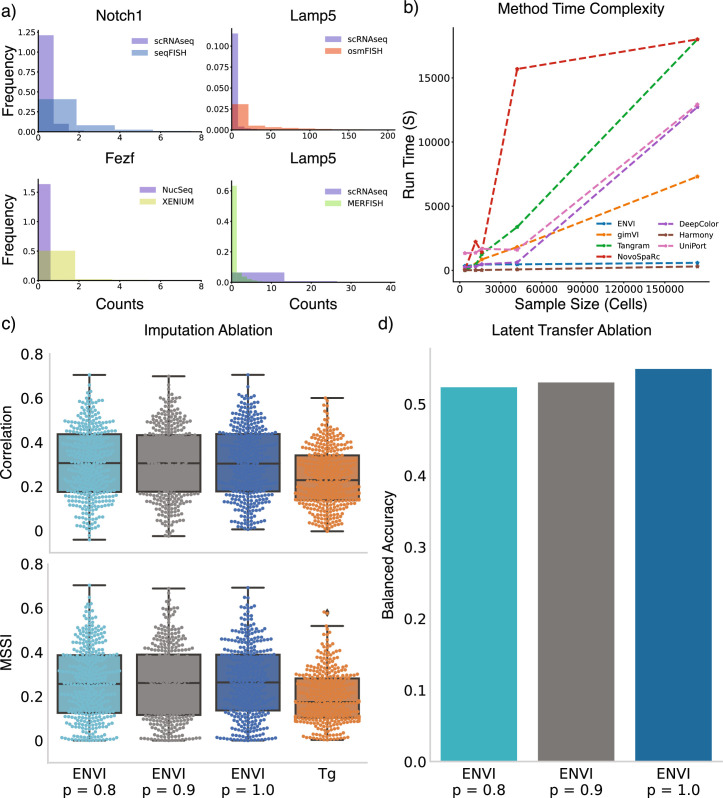
Modality-specific data features, run times and impact of sparsity on data integration. **a**, Examples of three genes that exhibit very different expression distributions between four spatial datasets and their matching scRNA-seq data. **b**, Run time of spatial and single-cell integration methods on real datasets of different sizes. Programs were manually terminated at 5 h (18,000 s). **c**, Benchmarking ENVI imputation of the full embryogenesis seqFISH and scRNA-seq dataset against 80% and 90% subsampled versions, as well as Tangram (Tg) on the full dataset as reference. Boxes and lines represent interquartile range (IQR) and median, respectively; whiskers represent ±1.5 x IQR. **d**, Ability of ENVI to transfer cell-type label information from scRNA-seq to spatial data (datasets as in **c**). ENVI retains cell-type information in the integrated latent even starting from sparser data.

Sequencing and spatial technologies measure different parameters and produce different data distributions and dynamic ranges (Extended Data Fig. 2a). ENVI takes this into account by marginalizing technology-specific effects on expression, augmenting the standard variational autoencoder (VAE) by adding an auxiliary binary neuron to the input layers of encoding and decoding networks for each modality. Moreover, ENVI parameterizes each modality with different probabilistic distributions, modeling single-cell data with a negative binomial by default to account for dropout^36^, and spatial data with a Poisson by default to reflect the high capture rate of fluorescence in situ hybridization (FISH)-based technologies^3^. ENVI thus integrates, imputes and reconstructs spatial context with a single end-to-end model, using deep learning for high-dimensional regression and variational inference for optimal integration of scRNA-seq and spatial data. The method scales to atlas-size datasets including millions of cells with constant time computational complexity (Extended Data Fig. 2b) while being robust to technology-specific artifacts, such as data sparsity (Extended Data Fig. 2c,d and Methods).

### ENVI imputes spatial patterns underlying gastrulation

**Fig. 2:**
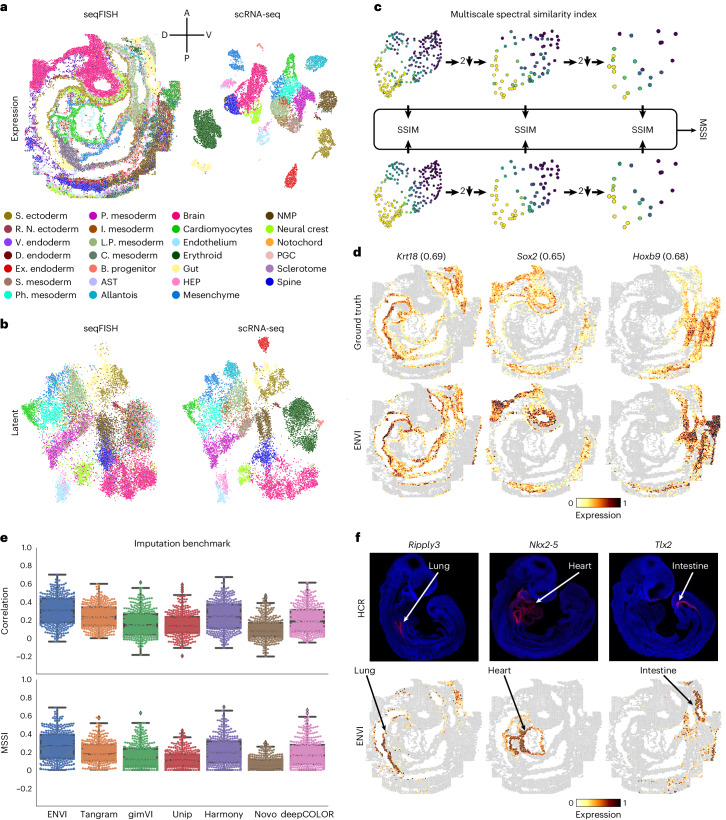
ENVI accurately recovers the expression of embryonic genes not imaged by multiplexed FISH. **a**, seqFISH^37^ image of an E8.75 mouse embryo sagittal section (left) and UMAP embedding of matched scRNA-seq data^38^ at E8.5 (right), both colored by major cell type compartment. **b**, UMAPs of ENVI latent embedding learned from mouse embryo data. Cells from seqFISH (left) and scRNA-seq (right) data are colored as in **a**. Average batch silhouette score bASW = 0.86 (Methods). **c**, Schematic of MSSI computation (Methods) for comparing two spatial expression profiles. Each profile is iteratively downsampled using spectral pooling on the cell proximity graph, and the SSIM is computed at each scale. MSSI is a weighted geometric mean of the SSIM computed at five scales, providing a spatially aware similarity metric on a scale from 0 to 1. **d**, seqFISH measurement in log counts (ground truth) and ENVI imputation for three withheld genes, marking endoderm (*Krt18*), neural stem (*Sox2*) and posterior section (*Hoxb9*) cells. MSSI values for each gene appear in parentheses. **e**, Pearson correlation and MSSI scores between log of seqFISH and imputed expression across all genes predicted from five-fold cross-validation, comparing four algorithms run with default parameters (Methods). Novo, NovoSpaRc; Tg, Tangram; Unip; uniPort. Boxes and lines represent interquartile range (IQR) and median, respectively; whiskers represent ±1.5× IQR. In order, MSSI\Pearson correlation *P* values (one-sided *t*-test, *n* = 351) are: $$4.45\cdot {10}^{-11}$$$$\backslash 4.75\cdot {10}^{-9}$$, $$3.45\cdot {10}^{-58}$$$$\backslash \,3.02\cdot {10}^{-94}$$, $$1.44\cdot {10}^{-76}\backslash 6.68\cdot {10}^{-93},$$$$\,4.83\cdot {10}^{-18}\backslash 3.44\cdot {10}^{-50}$$, $$4.12\cdot {10}^{-79}$$$$\backslash 15.47\cdot {10}^{-85}$$ and $$6.48\cdot {10}^{-32}$$$$\backslash 4.51\cdot {10}^{-69}$$. **f**, ENVI imputation (bottom) of organ marker genes not profiled by seqFISH: *Ripply3* (lung), *Nkx2-5* (heart) and *Tlx2* (intestine). Imputed expression matches associated organs and is validated by whole-mount HCR in situ (top). AST, anterior somitic tissue; B., blood; C, caudal; D., definitive; Ex., extraembryonic; HEP, hematoendothelial progenitor; I., intermediate; L.P., lateral plate; Novo, NovoSpaRc; P., paraxial; PGC, primordial germ cell; Ph., pharyngeal; R.N., rostral neuro; S. Ectoderm, surface ectoderm; S. Mesoderm, somitic mesoderm; Unip, uniPort; V., visceral.

We used ENVI to analyze a 350-gene sequential FISH (seqFISH)^37^ of mouse organogenesis at embryonic day 8.75 (E8.75) and matched scRNA-seq dataset^38^ at E8.5 (Fig. 2a). Unlike the discrete layers of adult brain tissue^3,39,40^ that dominate current spatial transcriptomic datasets, cells in developing embryos undergo rapid proliferation, differentiation and movement to create complex patterns and spatial gradients, presenting a challenging context for performance assessment. The most basic evaluation of any embedding-based data integration method is how well data across technologies co-embed, because this is critical for successful information transfer between modalities. The embedding learned by ENVI correctly maps major cell types to the combined latent space (Fig. 2b), as measured by average batch silhouette score^41^ (Methods).

Current FISH-based technologies only quantify the expression of hundreds of genes^12,37,40^, prompting the development of algorithms to impute the spatial patterns of unmeasured genes^9,25,26,42,43^. Previous studies^9,25,44^ used Pearson correlation and mean squared error between imputed and ground truth expression to evaluate the quality of imputation. However, both metrics are computed on a per-cell basis and ignore spatial context. To evaluate concordance between spatial patterns, we developed the multiscale spectral similarity index (MSSI), a metric that can capture similarity between spatial patterns by taking cell–cell proximity into account (Fig. 2c and Methods). MSSI borrows from the multiscale structure similarity index measure (MS-SSIM)^45^, a spatial pattern similarity metric widely used in computer vision that iteratively subsamples an image and assesses similarity at multiple resolutions. Our MSSI metric uses a cell–cell neighbor graph based on spatial proximity to generate a series of images at progressively lower resolutions by aggregating proximal cells and then applies SSIM to compare similarity at each resolution. MSSI is, thus, a spatially aware similarity metric that uses full count matrices and incorporates patterning at the cellular rather than pixel level and has multiple use cases, such as comparing the similarity of different gene expression patterns^46^.

**Extended Data Fig. 3:**
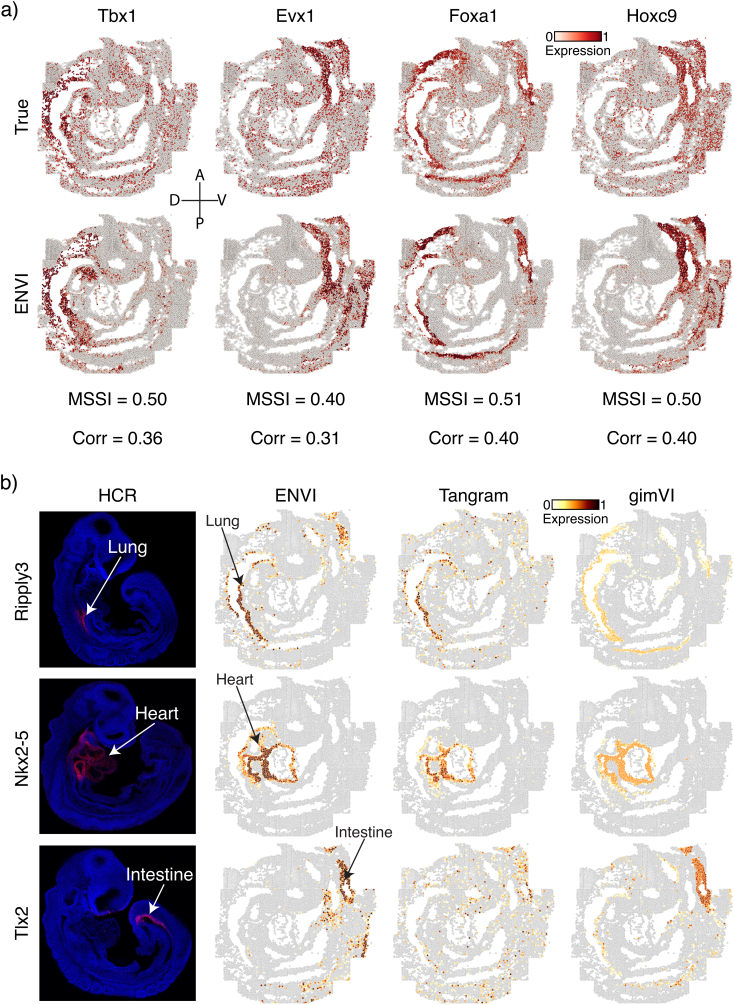
ENVI accurately infers embryogenesis genes missing from seqFISH data. **a**, Imputed expression of withheld genes from the seqFISH embryogenesis dataset^37^ (bottom) compared to true (measured) expression (top), with corresponding MSSI and Pearson correlation reconstruction (Corr) scores. **b**, HCR images of *Ripply3*, *Nkx2-5* and *Tlx2* and their imputation values according to ENVI, Tangram and gimVI. Organs marked by each gene are noted on the HCR and seqFISH images.

We used five-fold cross-validation (Methods) to compare ENVI imputation with measurements of held-out genes using both MSSI and Pearson correlation. The imputed expression of representative genes with clear spatial expression in endoderm (*Krt18*), neural stem (*Sox2*) and posterior section (*Hoxb9*) was visually similar to ground truth (Fig. 2d) and expressed in the correct organ. We found that some genes with correctly predicted organ-specific expression have high MSSI score but low Pearson correlation, supporting the importance of a spatially aware metric (Extended Data Fig. 3a).

We compared ENVI against Tangram^9^, gimVI^25^ and uniPort^47^, which were recently shown to outperform other integration methods^44^; NovoSpaRc^26^, because it uses fused optimal transport to explicitly model spatial context; deepCOLOR^48^, because it uses a deep generative model; and Harmony^49^, for its widespread use as a batch correction method^50^. ENVI significantly outperforms all other methods based on both MSSI and Pearson correlation (Fig. 2e).

Finally, we evaluated ENVI’s ability to impute genes beyond the 350-gene panel by assessing canonical markers of the developing lung (*Ripply3*)^51^, heart (*Nkx2-5*)^52^ and intestine (*Tlx2*)^53^. The expression of all three genes was validated as organ specific at E8.75 (before organ formation) by in situ hybridization chain reaction (HCR) imaging^54^ and was correctly imputed by ENVI (Fig. 2f). By contrast, Tangram and gimVI predicted weaker expression in the relevant region and anomalous expression beyond the organ (Extended Data Fig. 3b).

### ENVI ascribes spatial patterns to single-cell genomics data

In addition to gene imputation, ENVI can uniquely project spatial information onto dissociated cells profiled by scRNA-seq, by using its second decoder to reconstruct COVET matrices from the latent space. This approach can use limited spatial profiling data to confer spatial context onto the millions of cells in single-cell atlases. COVET represents gene–gene covariation between neighboring cells; thus, beyond deducing the cell type of neighbors, it can also infer their gene expression.

**Fig. 3:**
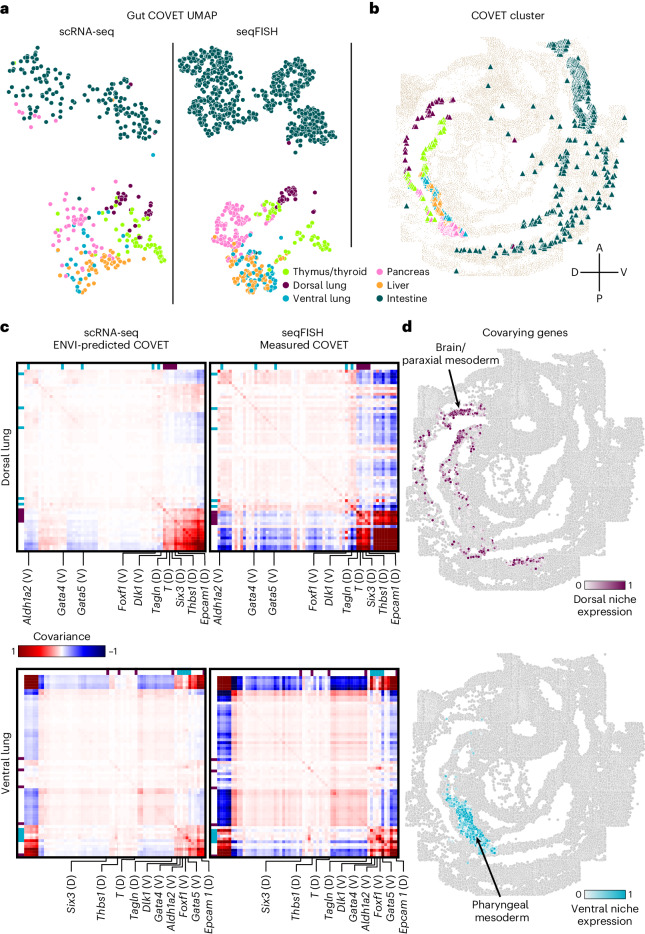
ENVI confers spatial context to single-cell samples from mouse gut organogenesis. **a**, COVET UMAP of gut tube cells. Each cell is represented by its directly measured (seqFISH) or ENVI-inferred (scRNA-seq) COVET, embedded in 2D coordinates by UMAP using the AOT metric. Cells are colored by emerging organ. **b**, seqFISH endoderm cells, colored by organ (based on label transfer from scRNA-seq), delineating spatial regions of the gut tube and showing coherence of organ assignments in distinct spatial niches. **c**, Average ENVI-imputed (scRNA-seq, left) or empirically measured (seqFISH, right) COVET matrices of dorsal (top) and ventral (bottom) lung cells from scRNA-seq (left) and seqFISH (right) datasets. Labeled gene modules (top row of each matrix) are uniquely present in either dorsal (purple) or ventral (cyan) lung COVET representations. **d**, Mean expression of the uniquely covarying genes in either dorsal (top) or ventral (bottom) lung COVET matrices in **c**. Only mesoderm cells surrounding the endoderm gut tube (but not part of it) are colored.

To demonstrate this ability, we used the mouse embryo dataset and focused on the gut tube, which generates the thymus, thyroid, lung, liver, pancreas, small intestine and colon in a stereotypical anterior-to-posterior sequence. Although E8.75 gut tube cells are anatomically indistinguishable, spatially delimited expression reveals that precursors are poised for their organ fates^55^. We computed COVET matrices for (measured) seqFISH data and used ENVI to infer COVET matrices for scRNA-seq data and then applied the AOT metric to co-embed matrices from both modalities (Fig. 3a). The rich transcriptional information in scRNA-seq data facilitated the assignment of endodermal organ identity to these cells^55^, and ENVI’s highly concordant co-embedding allowed for label transfer to those cells measured with seqFISH, as confirmed by anatomical localization; thymus and thyroid cells fall into the most anterior ventral gut tube, followed by dorsal and ventral lung clusters and, finally, intestine (Fig. 3b).

Using only endodermal scRNA-seq data, we plotted the average COVET matrices of dorsal and ventral lung and observed that these closely match empirical matrices computed from the seqFISH data (Fig. 3c). These COVET matrices infer modules of covarying genes in the niche environment and notably include genes expressed by adjacent mesodermal cells, which are known to provide spatial patterning cues to the endoderm^56^. To validate these inferred gene modules, we used the seqFISH data from mesodermal cells proximal to the gut tube (ignoring endodermal cells) and found that average ventral COVET gene expression is enriched in the ventral pharyngeal mesoderm, whereas average dorsal COVET gene expression is enriched in the dorsal brain and paraxial mesoderm (Fig. 3d). Our observations validate the predicted dorsal and ventral subdomains within the gut tube and demonstrate that ENVI can identify biologically important signaling originating from cells that were not sampled directly.

**Extended Data Fig. 4:**
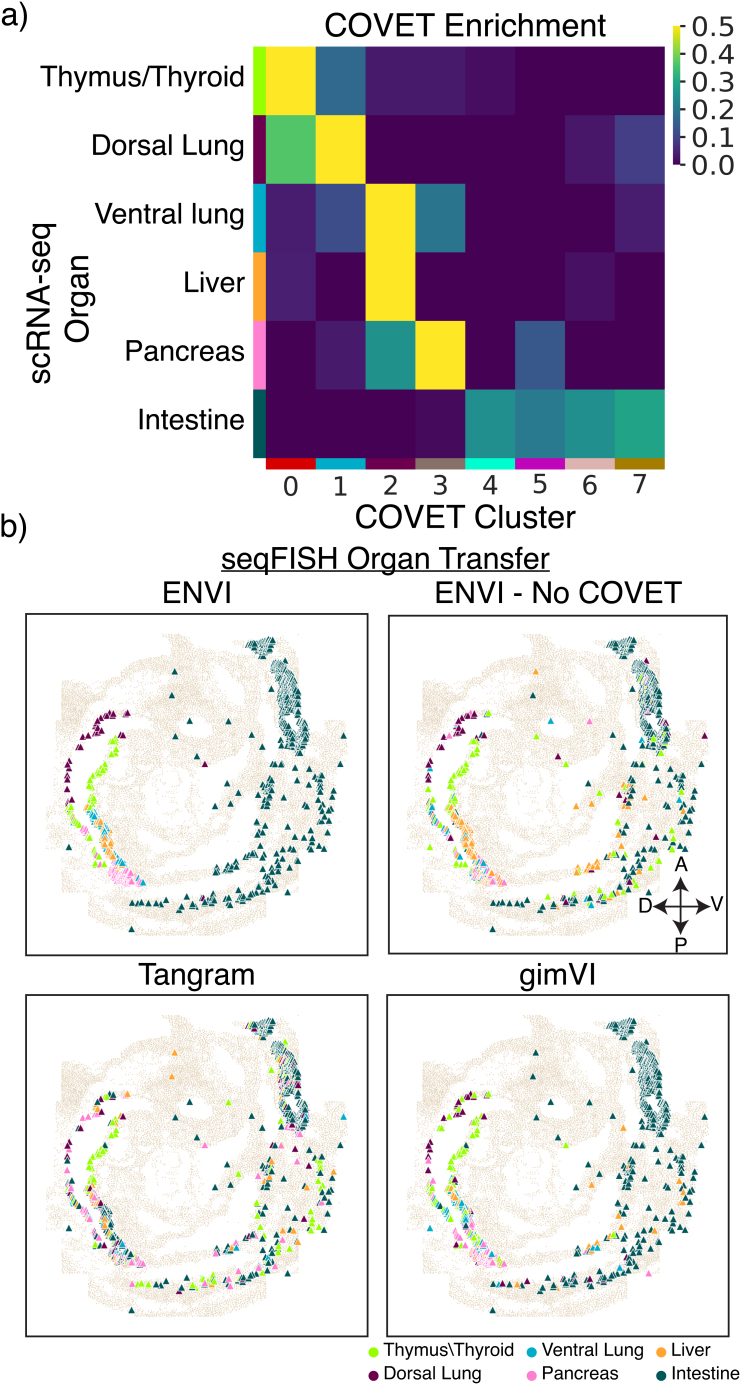
The use of COVET spatial covariance improves cell-type assignment. **a**, Proportion of scRNA-seq gut tube cells in each organ (row) that fall into each COVET cluster (column), arranged from anterior to posterior. **b**, Assignment of developing organs to seqFISH gut tube cells via ENVI COVET space, latent space of ENVI when trained without COVET, gimVI latent space and Tangram cell-type mapping.

**Extended Data Fig. 5:**
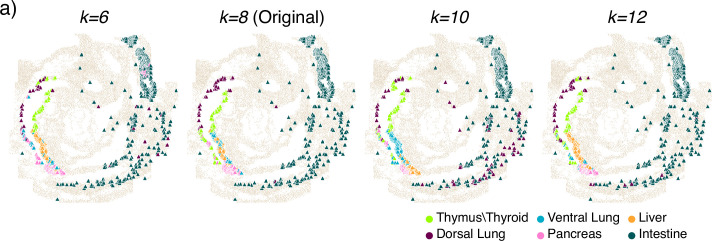
ENVI is robust to variation in COVET neighborhood size. Transfer of organ labels onto seqFISH gut tube cells according to independent instances of ENVI, each trained according to COVET representations based on a different number of nearest spatial neighbors (*k*). Spatial context predictions remain robust across *k* values.

Using spatial covariance can also markedly improve the simpler task of labeling organ identity for cells from the spatial modality, as seqFISH measures fewer genes and cells than scRNA-seq and is, thus, more difficult to label. Labeling scRNA-seq cells from the gut tube with organ-specific gene sets^55^ (Methods) revealed an almost one-to-one matching between organ precursors and COVET clusters, whereas ENVI without COVET failed to generate accurate labels, and alternative approaches were even less accurate (Extended Data Fig. 4). ENVI-based label transfer is also robust to variation in neighborhood size when computing COVET (Extended Data Fig. 5).

### ENVI learns spatial gradients from single-cell data

**Fig. 4:**
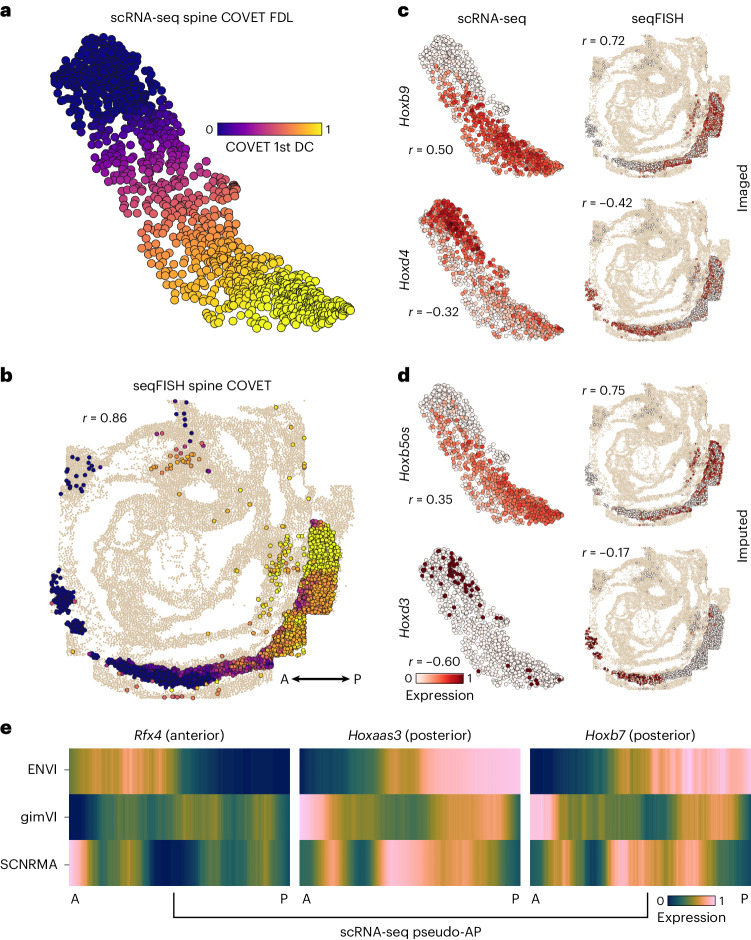
ENVI maps continuous spatial gradients in spine development from single-cell and spatial data. **a**, FDL based on ENVI-predicted COVET matrices of scRNA-seq spinal cord cells and their NMP precursors, colored by the first COVET diffusion component (pseudo-AP). FDL and DCs were calculated using both the seqFISH and scRNA-seq COVET matrices. **b**, seqFISH NMPs and spine cells colored by the first COVET DC, which traverses the AP axis. Pearson correlation (*r*) is calculated between pseudo-AP and true AP. **c**, Expression of *Hoxd4* (anterior) and *Hoxb9* (posterior) markers in NMPs and spine cells from scRNA-seq and seqFISH data, recapitulating expected AP localization. **d**, Same as **c**, colored by ENVI-imputed *Hoxb5os* (posterior) and *Hoxd3* (anterior) expression. These markers were not imaged. **e**, scRNA-seq expression of canonical AP axis markers in NMPs and spine cells ordered along pseudo-AP axis according to different integration methods. SCNRMA, Scanorama.

**Extended Data Fig. 6:**
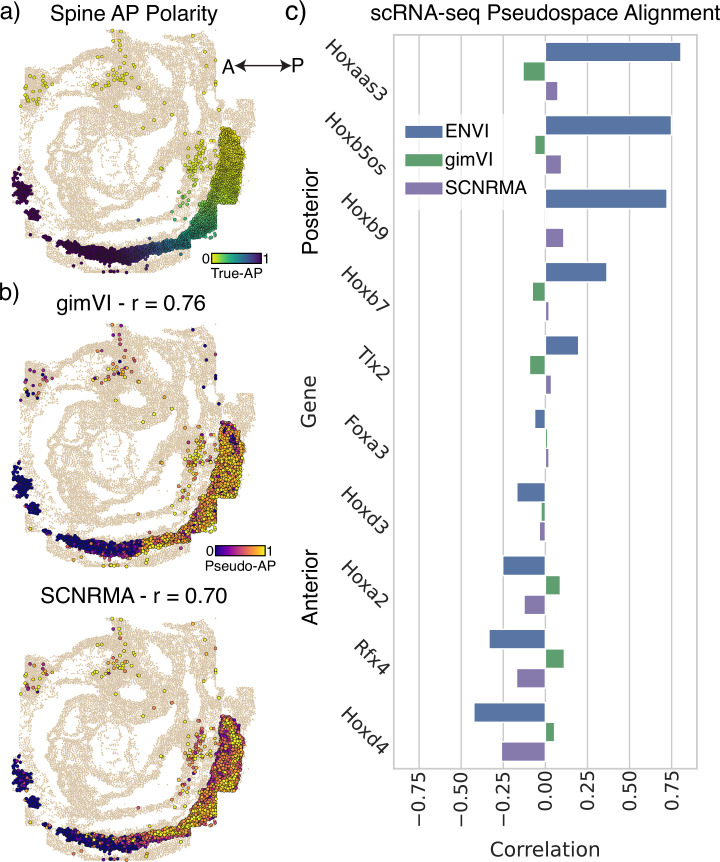
ENVI reliably recovers the AP axis during spine development. **a**, Spine and NMP cells from seqFISH data, colored by AP polarity calculated from the first DC of their spatial coordinates. **b**, Pseudo-AP of seqFISH spine and NMP cells from DC analysis of gimVI and Scanorama. Values denote Pearson correlation with the true AP axis. **c**, Pearson correlation of ENVI COVET, gimVI and Scanorama pseudo-AP of spine and NMP scRNA-seq cells, for five canonical posterior markers (higher is better) and anterior markers (lower is better). Pseudo-AP axis is based on the DC best aligned with true depth (DC 1, 2 and 3 for ENVI, gimVI and Scanorama, respectively).

Although the gut tube is defined by relatively discrete primordial organs, many processes—such as the specification of spinal cord cells and their precursors, the neuromesodermal progenitors (NMPs), along the anteroposterior (AP) axis—are organized by continuous spatial gradients^57^. To highlight ENVI’s ability to model gradients, we co-embedded empirical seqFISH COVET matrices with ENVI-inferred scRNA-seq COVET matrices for NMPs and spine cells using a force-directed layout (FDL)^58^ and calculated their diffusion components (DCs) (Fig. 4a). The first DC is highly congruent with the AP axis (Pearson correlation = 0.86), demonstrating that COVET can capture gradual spatial trends (Fig. 4a,b and Extended Data Fig. 6a). As the COVET DC is calculated from both seqFISH and scRNA-seq datasets, we can use it to assign AP pseudo-coordinates to NMPs and spine cells from scRNA-seq data.

The first COVET DC correctly reveals that scRNA-seq cells are enriched for *Hoxd4* (refs. ^55,59^) (anterior) and *Hoxb9* (ref. ^60^) (posterior) markers in their respective domains, consistent with seqFISH expression in NMPs and spine cells (Fig. 4c). Furthermore, ENVI correctly mapped high expression of *Hoxd3* (ref. ^59^) (anterior) and *Hoxb5os*^61^ (posterior) markers to scRNA-seq cells in their corresponding AP domains, demonstrating that ENVI spatial modeling extends to genes that are not imaged (Fig. 4d). Conversely, ENVI-imputed *Hoxb5os* and *Hoxd3* expression for the seqFISH data mirrors the predicted spatial context of the scRNA-seq data.

We found that the major axis of variation (first DC) between the COVET matrices that model the niche reflects the spatial organization of the tissue; ordering NMPs and spine cells along DC 1 recovers a pseudo-AP axis that can be used to visualize predicted expression trends^57^ (Fig. 4e). Similar analysis using the gimVI latent space and Scanorama^62^ integration (Methods) led to inferior alignment with the true AP axis (Extended Data Fig. 6b), despite selecting the gimVI and Scanorama DCs most correlated (*r* = 0.76 and *r* = 0.7070, respectively) with true AP polarity. This slightly lower correlation with the AP axis propagates into more pronounced inaccuracies in expression patterns; only ENVI correctly derived expected AP trends for *Rfx4* (ref. ^63^), *Hoxaas3* (ref. ^61^) and *Hoxb7* (ref. ^64^) (Fig. 4e). More generally, both anterior and posterior canonical markers are more correlated (or anti-correlated) with ENVI COVET pseudo-AP than with axes defined by gimVI and Scanorama (Extended Data Fig. 6c). ENVI can, thus, correctly uncover AP polarity within single-cell NMPs and spine cells and correctly place them along this spatial axis.

### ENVI delineates tissue-scale patterning in the motor cortex

Although data integration is typically evaluated on abundant neural cell types that dominate spatial regions, we challenged ENVI to recover rare cell types. Somatostatin (*Sst)*-expressing interneurons are a cardinal class of inhibitory neurons in the cortex^65^ that are implicated in Alzheimer’s disease and depression^66^ and encompass substantial diversity^67–69^. Although we know that *Sst* interneurons influence their environment, their localization and its relationship to function and transcriptional states have not been fully explored.

**Fig. 5:**
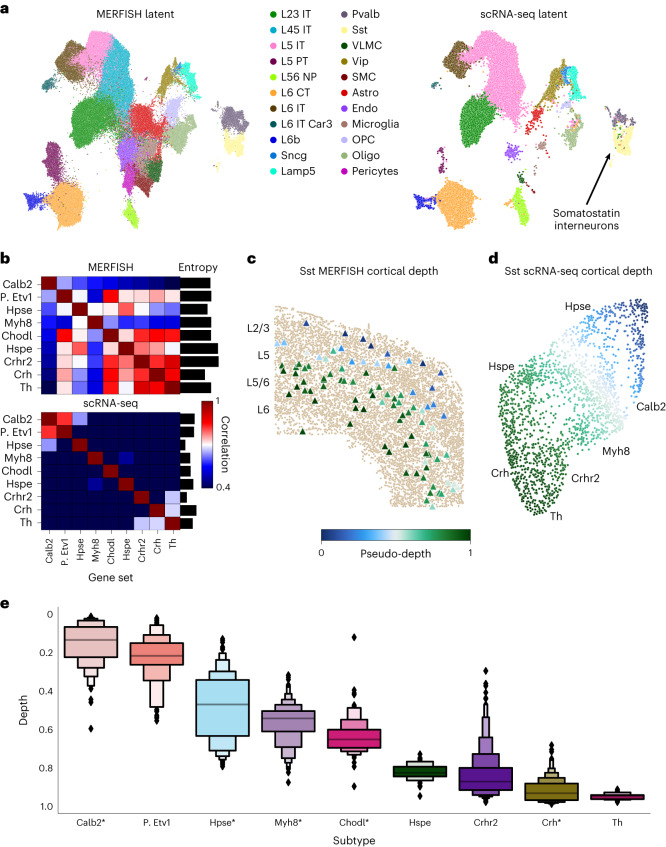
ENVI predicts the cortical localization of *Sst* interneuron subtypes. **a**, ENVI latent embedding of motor cortex MERFISH (left) and scRNA-seq (right) data, labeled by cell types from Zhang et al.^40^ and Yao et al.^70^, and highlighting *Sst* interneurons. Latent integration bASW = 0.78. OPC, oligodendrocyte precursor cell. **b**, Correlation between subtype-specific gene sets (Supplementary Table 3) in *Sst* interneurons from the MERFISH and scRNA-seq datasets. Bar plots mark the entropy of overlap for each subgroup (higher values denote poorer delineation into distinct subtypes). **c**, *Sst* interneurons from MERFISH data, colored by first COVET DC representing pseudodepth. L2/3 to L6 indicate approximate location of cortical layers. **d**, FDL of scRNA-seq COVET for all *Sst* interneurons, colored by first COVET DC. COVET FDL and DCs are calculated based on both scRNA-seq and MERFISH datasets. Specific subtypes, labeled by Yao et al.^70^, are marked. **e**, COVET DC-predicted cortical depth of scRNA-seq *Sst* interneurons, grouped by labeled subtype. *, cell types imaged in Wu et al.^71^; all empirical depths match ENVI predictions. In the box plot, line is median; first boxes include the 50th percentile; and each successive pair of boxes outward contain half of the remaining data.

**Extended Data Fig. 7:**
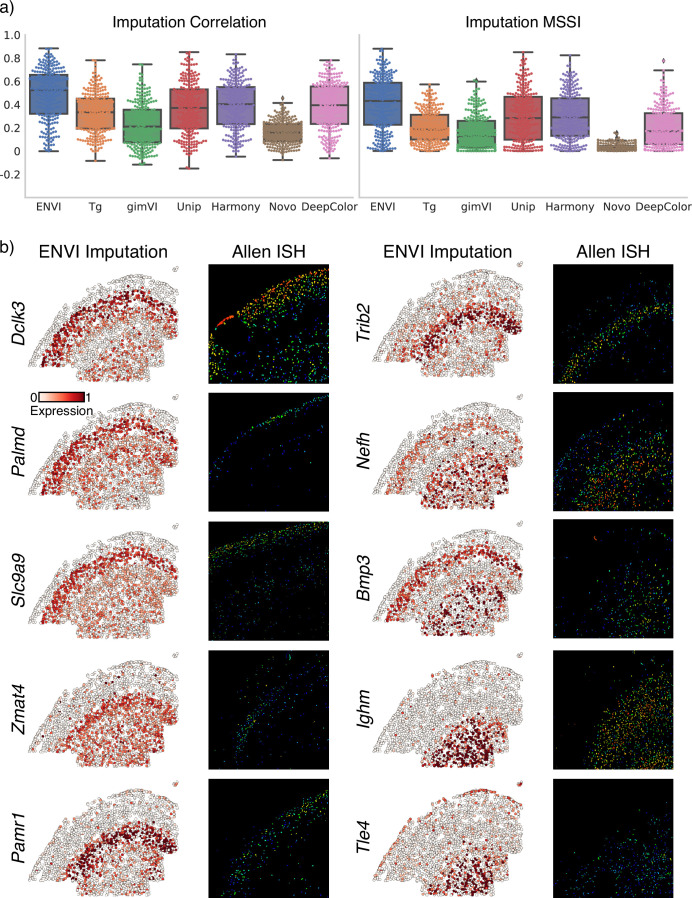
ENVI extends cortical tissue gene expression to the entire genome. **a**, Five-fold cross-validation of imputation based on a 252-gene MERFISH dataset from the primary motor cortex^40^. Boxes and lines represent IQR and median, respectively; whiskers represent ±1.5 x IQR. Comparison of MSSI\Pearson correlations between ENVI and other methods (one-sided t-test, *n* = 252) generates p-values, from left (Tangram) to right (deepC), of $4.67\cdot {10}^{-29}\backslash 1.80\cdot {10}^{-19}$, $1.62\cdot {10}^{-72}\backslash 7.29\cdot {10}^{-66}$, $4.70\cdot {10}^{-36}\backslash 1.72\cdot {10}^{-50}$, $5.25\cdot {10}^{-28}\backslash 4.10\cdot {10}^{-41}$, $5.23\cdot {10}^{-72}\backslash 1.35\cdot {10}^{-86}$, $1.87\cdot {10}^{-64}\backslash 2.38\cdot {10}^{-66}$. **b**, ENVI imputation of genes selected due to their clear in situ hybridization profiles in the Allen Brain Atlas (mouse.brain-map.org), projected onto the MERFISH data, with corresponding ISH expression in the motor cortex. Novo, NovoSpaRc; deepC, deepCOLOR.

To localize *Sst* interneurons, we analyzed the scRNA-seq (71,183-cell) and 252-gene MERFISH (276,556-cell) atlases of the motor cortex of the Brain Initiative Cell Census Network (BICCN)^40,70^. ENVI outperformed all other tested methods in both speed (training on this large atlas in minutes) and imputation (Extended Data Figs. 2b and 7), and it successfully co-embedded the 22 BICCN-annotated coarse cell types (Fig. 5a). Notably, only scRNA-seq data can distinguish the nine distinct *Sst* subpopulations, as the MERFISH panel lacks requisite marker genes (Fig. 5b and Methods).

Using ENVI-imputed COVET matrices, we mapped *Sst* interneurons labeled in the scRNA-seq dataset to their location within the cortex. We found that—despite being interspersed throughout the cortex, where cell types, such as excitatory neurons, dominate—the first DC of COVET matrices is highly correlated with cortical depth, thus defining a ‘pseudodepth’ axis (Fig. 5c,d), and that *Sst* subtypes are predicted to stratify by depth (Fig. 5e). Molecular imaging by genetic strategies targeting *Sst* subtypes^71^ validates a number of our predictions, including the localization of *Calb2* interneurons to the L2/3 layers and *Crh* interneurons to L6. Beyond these, ENVI predicted the cortical depth of many subtypes identified in the scRNA-seq atlas with unknown localization. For example, it placed *Sst* interneurons expressing high levels of the neurotransmitter metabolism gene *Tyrosine hydroxylase* in the deep L6 layer, as might be expected, suggesting that ENVI can articulate the interplay between transcriptional state and microenvironment.

**Extended Data Fig. 8:**
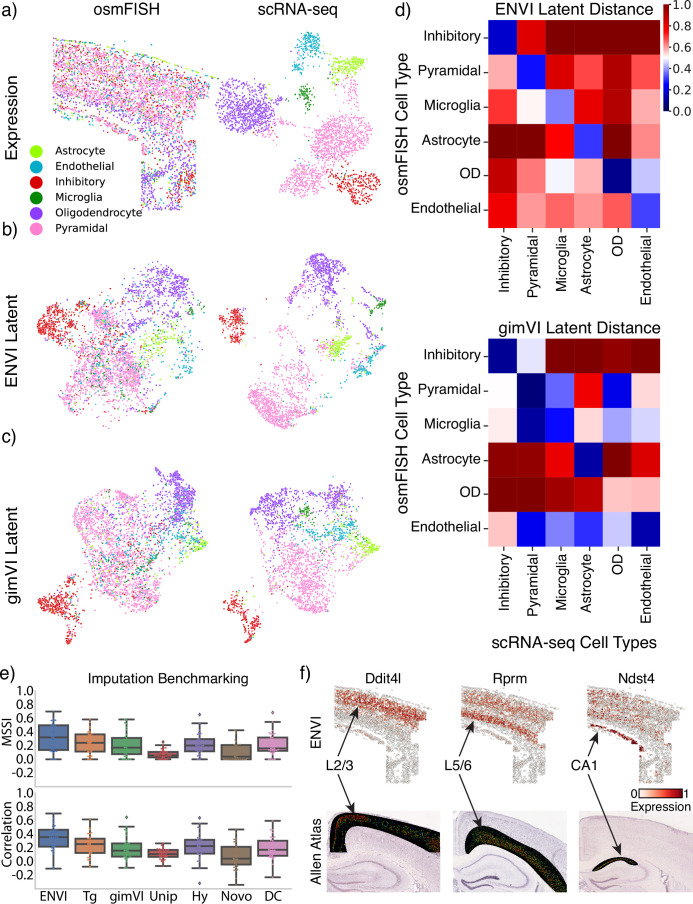
ENVI integrates osmFISH and scRNA-seq data from the somatosensory cortex. **a**, osmFISH with segmented cells and UMAP visualization of scRNA-seq datasets of the mouse somatosensory cortex, colored by cell types as annotated in Codeluppi et al.^3^. **b**, UMAP visualizations of the ENVI integrated latent embedding of the osmFISH and scRNA-seq modalities, colored by cell types as in **a**. Latent integration score; bASW = 0.62. **c**, Same as **b**, but with latent embeddings from gimVI. **d**, Normalized distance between the center-of-mass of each cell type according to the ENVI and gimVI latent embeddings. **e**, Benchmarking of imputation based on leave-one-out training, evaluated by Pearson correlation and MSSI on a 33-gene osmFISH dataset of the somatosensory cortex^3^. Boxes and lines represent IQR and median, respectively; whiskers represent ±1.5 x IQR. In order, MSSI\Pearson correlation p-values (one-sided t-test, *n* = 33) are: $1.41\cdot {10}^{-4}\backslash 6.69\cdot {10}^{-10}$, $9.03\cdot {10}^{-4}\backslash 2.64\cdot {10}^{-6}$, $5.25\cdot {10}^{-9}\backslash 2.03\cdot {10}^{-10}$, $1.21\cdot {10}^{-3}\backslash 1.62\cdot {10}^{-5}$, $4.34\cdot {10}^{-10}\backslash 1.43\cdot {10}^{-9}$, $7.11\cdot {10}^{-4}\backslash 9.08\cdot {10}^{-6}$**. f**, ENVI-imputed expression of unimaged cortical markers *Ddit4l* (L2/3), *Rprm* (L5/6) and *Ndst* (Hippocampus, CA1) (top) and corresponding expression in the Allen Brain Atlas (mouse.brain-map.org) (bottom). Tg, Tangram; Hy, Harmony; Novo, NovoSpaRc; DC, deepCOLOR.

ENVI can also capture spatial patterns within the cortex from datasets that include only a few imaged genes. Applied to a 33-gene osmFISH and matched scRNA-seq dataset of the somatosensory cortex^3^, ENVI successfully integrated the small datasets (fewer than 10,000 cells combined) into a unified embedding (Extended Data Fig. 8a,b) and outperformed alternative methods in cell type resolution and spatial gene imputation (Extended Data Fig. 8c–e). To determine whether ENVI can impute unimaged genes, we leveraged Allen Brain Atlas ground truth data for mouse brain cortex (https://mouse.brain-map.org/) and confirmed that ENVI correctly imputes layer-specific spatial expression for *Dti4l*, *Rprm* and *Ntst4* in the L2/3, L5/6 and CA1 regions, respectively (Extended Data Fig. 8f).

### ENVI integrates Xenium data on brain metastasis

**Fig. 6:**
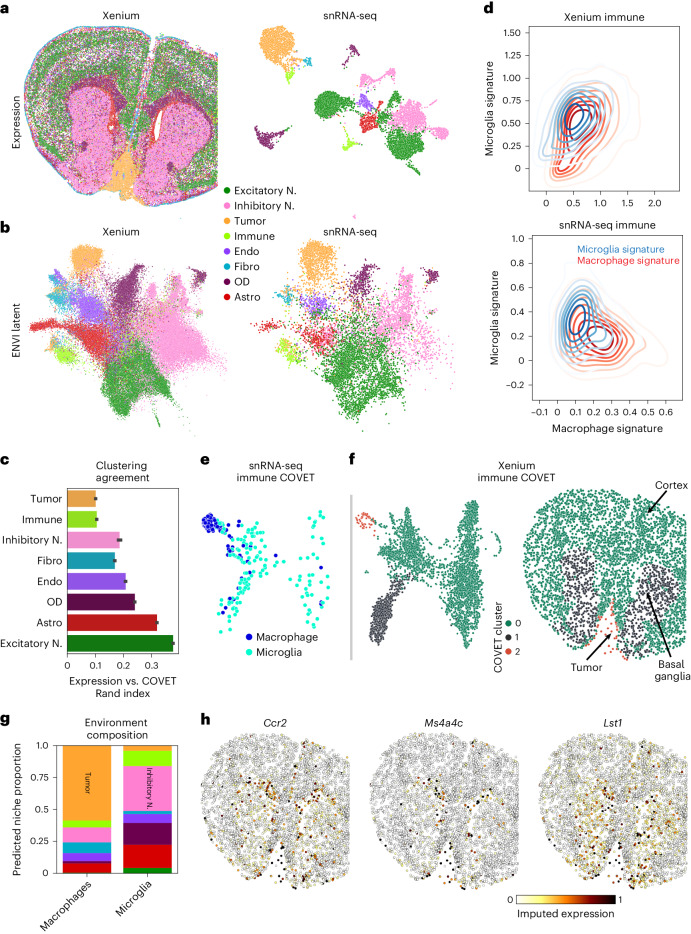
ENVI integrates Xenium and snRNA-seq data to localize neuroimmune cell types during metastasis. **a**, Xenium image and UMAP embedding of snRNA-seq data from mouse brain bearing a melanoma metastasis, colored by major cell type. **b**, UMAP embeddings of ENVI latent space showing cells from the spatial (left) and snRNA-seq (right) datasets. Similar cell types, including malignant cells, co-embed across modalities. bASW = 0.87. **c**, Average concordance of technical replicates between the expression of each cell type and its environment in the Xenium data, measured as ARI between *k*-means clusters of gene expression and COVET representation. Error bars, 95% confidence interval. **d**, Density plots of microglia and macrophage cell signature expression in immune-labeled cells from Xenium (top) and snRNA-seq (bottom) datasets. Only snRNA-seq data measure enough genes to separate cell types. **e**, UMAP embedding of the ENVI-predicted COVET representation of snRNA-seq immune cells, colored by subtype. **f**, COVET UMAP (left) and spatial coordinates (right) of Xenium immune cells, colored by COVET clusters representing major immune cell microenvironments: cortex (C0), basal ganglia (C1) and tumor (C2). COVET UMAP and clusters are calculated from both snRNA-seq and Xenium datasets. The vast majority (85%) of snRNA-seq macrophages were assigned to C2, predicting their localization to the tumor. **g**, Cell type fractions of ENVI-predicted environments for snRNA-seq macrophages and microglia, colored as in **a**, highlighting their predominant tumor and inhibitory neuron environments, respectively. **h**, ENVI-imputed macrophage infiltration marker genes on Xenium immune cell types are distinctly enriched within the metastasized tumor. Astro, astrocyte; Endo, endoderm; Fibro, fibroblast; OD, oligodendrocyte; N., neuron.

**Extended Data Fig. 9:**
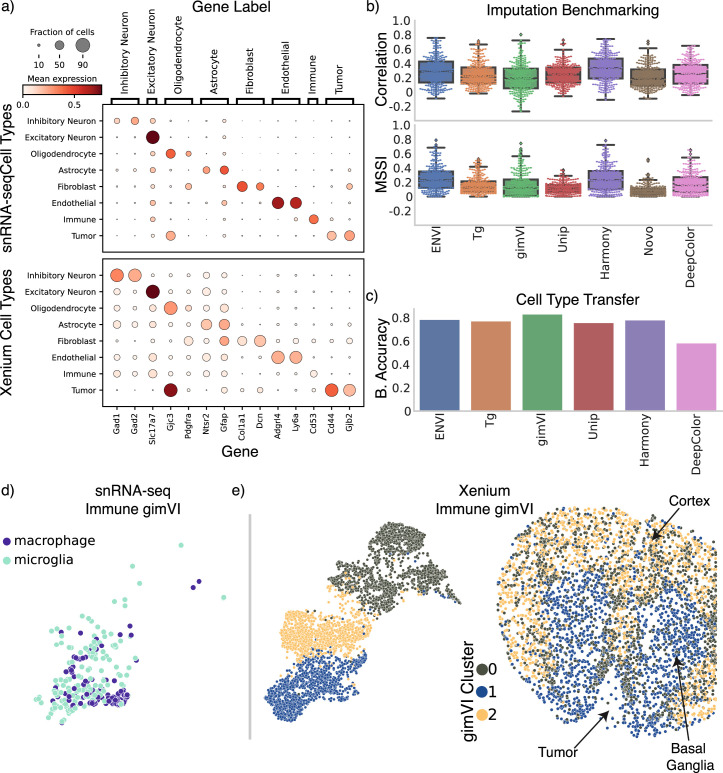
Cell type validation and extended benchmarking of ENVI on Xenium data. **a**, Expression of marker genes for each annotated cell type in the snRNA-seq and Xenium data. **b**, Extended imputation benchmarking of ENVI against uniPort (Unip), Harmony and deepCOLOR. Boxes and lines represent IQR and median, respectively; whiskers represent ±1.5 x IQR. In order, MSSI\Pearson correlation p-values (one-sided t-test, *n* = 243) are: $1.21\cdot {10}^{-11}\backslash 1.88\cdot {10}^{-3}$, $6.62\cdot {10}^{-16}\backslash 3.43\cdot {10}^{-13}$, $1.20\cdot {10}^{-33}\backslash 5.82\cdot {10}^{-7}$, $3.93\cdot {10}^{-1}\backslash 9.99\cdot {10}^{-1}$, $2.66\cdot {10}^{-45}\backslash 4.89\cdot {10}^{-19}$, $6.92\cdot {10}^{-12}\backslash 1.13\cdot {10}^{-4}$. **c**, Balanced accuracy for annotating Xenium cell types from snRNA-seq labels. ENVI, Tangram (Tg), gimVI, uniPort (Unip) and Harmony all perform similarly. **d**, UMAP of gimVI latent space of snRNA-seq immune cells, colored by subtype. **e**, gimVI latent UMAP and PhenoGraph clusters of Xenium immune cells. UMAP and clusters are calculated using both Xenium and snRNA-seq immune cells. Most microglia (68%) and macrophages (90%) are assigned to cluster C1, preventing clear association between subtype and microenvironment.

Leptomeningeal metastasis (LM) is a lethal condition in which distant tumor cells spread into the fluid-filled space surrounding the central nervous system^72,73^. The poor understanding of interactions among tumor, immune and underlying brain parenchyma cells limits the discovery of therapeutics. We used the Xenium platform (10x Genomics)^16^ to perform in situ hybridization (ISH) of 243 genes in a mouse model of melanoma LM^74^ and also sequenced cells from an adjacent section using a custom single-nucleus RNA sequencing (snRNA-seq) protocol (Methods) that we developed by optimizing RNA extraction from formalin-fixed paraffin-embedded (FFPE) samples, followed by 10x Genomics Flex probe-based library preparation. We separately clustered and annotated the spatial and single-cell samples into major cell types based on marker genes (Fig. 6a and Extended Data Fig. 9a). Even in this pathological context, ENVI performance with default parameters matches or exceeds competing methods on gene imputation (Extended Data Fig. 9b) and harmonizes the two datasets into a unified latent space (Fig. 6b and Extended Data Fig. 9c).

Our approach provides two representations of the Xenium data; we can visualize and cluster each cell based either on its gene expression or on its COVET matrix (representing the local niche). Measuring the agreement between clustering of the two representations reveals that, as expected, excitatory neuron expression depends strongly on spatial context, due to the association between distinct cortical layers and molecular markers^70^, whereas tumor and immune cell types show little concordance between expression and environmental context (Fig. 6c).

Melanoma LM interacts with two key immune populations: tissue-resident brain macrophages, known as microglia, and monocyte-derived macrophages that are recruited to the tumor lesion and colonize it from the periphery^74^. The snRNA-seq data clearly distinguish these myeloid subtypes based on curated gene sets^75^, whereas the Xenium brain panel lacks the markers to distinguish macrophages and microglia (Fig. 6d). To resolve where the subtypes localize, we co-embedded ENVI-imputed snRNA-seq COVET matrices with observed Xenium COVET matrices and clustered the data, revealing three distinct immune microenvironments consisting of cortical, basal ganglia or tumor cells (Fig. 6e,f). The snRNA-seq data enabled the labeling of cluster 2 as non-resident macrophage, and the Xenium data allowed us to visualize the localization of cells from this COVET cluster. Confirming known patterns of neuroimmune cell types^76–78^, most microglia were assigned to the basal ganglia and cortex, whereas most macrophages were localized to the tumor and its boundary. COVET allowed us to infer the niche composition for each immune cell in the snRNA-seq data, which corroborates that macrophages are found mainly near tumor cells, whereas microglia are found mainly near neurons and other glial cells (Fig. 6g).

**Extended Data Fig. 10:**
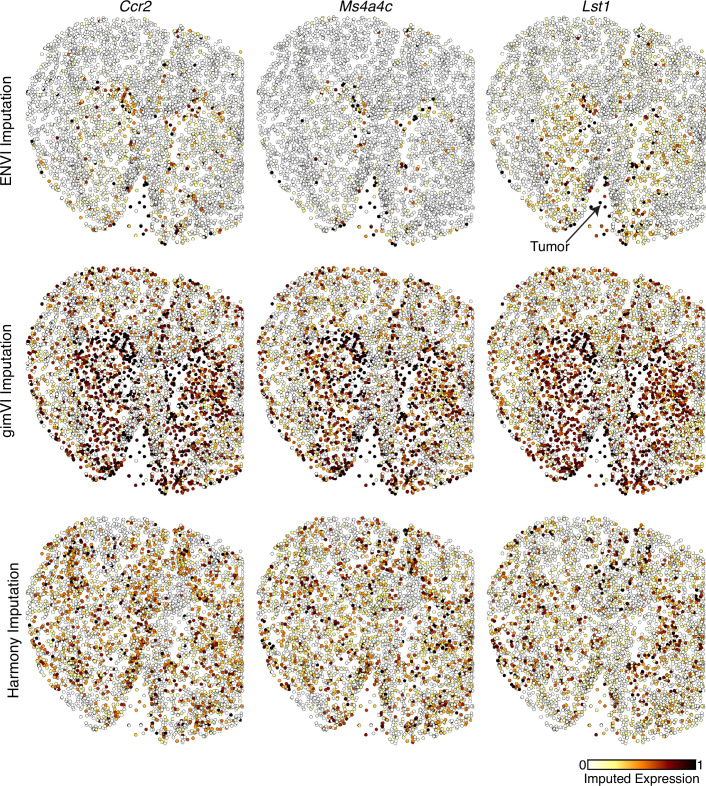
Imputation of tumor-infiltrating macrophage markers at the tumor-immune boundary. ENVI, gimVI and Harmony-based imputation of three tumor-infiltrating macrophage markers onto Xenium immune cells. Only ENVI correctly predicts the expected pattern of expression, showing enrichment in immune cells inside the tumor region.

Beyond localizing macrophages and microglia, ENVI can distinguish the transcriptional patterns of tumor-infiltrating macrophages from those on the boundary by imputing gene expression in the Xenium data (Fig. 6h). For instance, imputation of *Ccr2*, a chemokine receptor that recruits monocytes to the tumor and promotes their differentiation into tumor-associated macrophages^78^, was enriched in immune cells within the tumor and its vicinity. In contrast, clustering-based analysis of the gimVI latent on the immune cells does not clearly assign macrophages to a malignant microenvironment, and its gene imputation is also inaccurate, predicting that tumor infiltration genes are broadly expressed across the brain (Extended Data Fig. 9d,e). Harmony and gimVI also fail to localize infiltration marker expression to immune cells within the tumor (Extended Data Fig. 10).

## Discussion

ENVI robustly integrates scRNA-seq and spatial transcriptomics data, overcoming technical biases while retaining biological information. The algorithm provides superior performance for imputing missing gene expression in spatial modalities; it scales to millions of cells; and it has the distinctive ability to infer the spatial context of dissociated cells, even across multiple cell types in complex tissues.

ENVI’s capabilities rely on COVET as a representation of spatial niches. Although most spatial representations are based on discrete cell typing, COVET takes full advantage of the quantitative nature of gene expression data. The COVET matrix captures covariation between markers in a cell’s niche and uses optimal transport to derive a principled and quantitative model of cellular neighborhoods. COVET powers a shift from discrete cell type to continuous cell state paradigms and the discovery of continuous trends in spatial microenvironments.

ENVI performance is primarily driven by three factors: (1) deep Bayesian inference to regress out modality-related confounders while learning nonlinear relationships between genes and niches; (2) explicit modeling of the entire transcriptome from scRNA-seq data; and (3) direct incorporation of spatial context via COVET. Whereas current methods only learn the genes that overlap between scRNA-seq and spatial datasets, ENVI models all available information and does not rely on post hoc inference. This proves invaluable, as the ENVI model is imbued with both spatial context and full transcriptome information, allowing for reliable transfer of information between modalities.

The ENVI COVET space can correctly predict primordial organ niches from seqFISH and scRNA-seq data of mouse gastrulation, and COVET-based DC analysis can highlight continuous AP trends of both expression and environment in the developing spine. ENVI’s critical ability to confer spatial context onto dissociated single cells drives the inference of circuits of *Sst* interneuron subtypes in the motor cortex. Moreover, it provides an accurate representation of both discrete and diffuse signals in healthy and pathological tissue contexts, enabling spatial reasoning along the full transcriptome, including the spatial distinction of subtly different tumor and non-tumor-associated macrophage cell states in metastatic tissue.

One caveat is that the range of spatial factors can vary, whereas COVET is currently defined at a single scale set by the neighborhood size, *k*. Although COVET is relatively robust to small changes of *k*, larger differences may lead to different outcomes, and its value should be tuned to the spatial questions of interest.

## Methods

### Computational methods

#### MSSI

When comparing the spatial distribution of genes or markers across a tissue, it is imperative to have a robust metric that takes spatial structure into account. Although ubiquitous metrics, such as Pearson correlation, SSIM and root mean square error, can provide some insight, they lack spatial context (for example, cell–cell proximity or spatial patterns) and measure only per-cell discrepancy.

To devise a metric for spatial data, we borrowed the MS-SSIM^45^, a ubiquitous metric for the quality of image reconstruction, from computer vision. Given two images, MS-SSIM iteratively downsamples each image, creating an image pyramid^79^—a multiscale signal representation consisting of the same image at multiple resolutions. MS-SSIM returns a weighted geometric average of the standard SSIM scores between the two images at each scale of the pyramid. Standard SSIM for two images, *x* and *y*, is

$${\mathrm{SSIM}}(x,y)=l(x,y)\cdot c(x,y)\cdot s(x,y),$$

where

$$\begin{array}{l}l(x,y)\,=\,\displaystyle\frac{2{\mu }_{x}{{\rm{\mu }}}_{{\rm{y}}}+{(0.01\cdot M)}^{2}}{{\mu }_{x}^{2}+{\mu }_{y}^{2}+\frac{0.01}{M}},c(x,y)=\displaystyle\frac{2{\sigma }_{x}{\sigma }_{y}+{(0.03\cdot M)}^{2}}{{\sigma }_{x}^{2}+{\sigma }_{y}^{2}+{(0.03\cdot M)}^{2}},\\s(x,y)=\displaystyle\frac{{\sigma }_{xy}+\displaystyle\frac{{(0.03\cdot M)}^{2}}{2}}{{\sigma }_{x}{\sigma }_{y}+\displaystyle\frac{{(0.03\cdot M)}^{2}}{2}}\end{array}$$

*M* represents the maximum values between *x* and *y*; *μ*_*x*_ and *μ*_*y*_ are their average values; *σ*_*x*_ and *σ*_*y*_ measure how each varies; and *σ*_*xy*_ represents how much they covary. *l*(*x*,*y*), *c*(*x*,*y*) and *s*(*x*,*y*) are measures of ‘luminance’ (signal brightness), contrast and structure, respectively. Although SSIM is meant for images, it can also be calculated between any two vectors of similar sizes.

We introduce the MSSI as an adaptation of MS-SSIM to spatial transcriptomics that compares count matrices from segmented cells, rather than pixels, using a neighbor graph of spatially neighboring cells to capture structure. Intuitively, MSSI is a spectral analog of MS-SSIM; by rephrasing image coarsening to its graph-based counterpart, we can apply it to segmented cells and produce a multiscale, spatially driven score of expression reconstruction quality.

MSSI compares the expression profiles of two genes from a multiplexed image: $X={\{{x}_{i}\}}_{i=1}^{N}$ and $Y={\{{y}_{i}\}}_{i=1}^{N}$, where the index *i* enumerates segmented cells, and *x* and *y* can be either (1) two different genes or (2) a ground truth gene and its imputed value. In addition, the spatial coordinate of each cell is $D={\{{D}_{i}\}}_{i=1}^{N}$. We first compute the *k* nearest neighbor (kNN) graph *G*^1^ of segmented cells from ${\{{D}_{i}\}}_{i=1}^{N}$. To generate a subsampled version of the kNN graph, we use a graph coarsening algorithm^80^, which pools nodes together based on their connectivity pattern, similarly to how image downsampling groups pixels together (Fig. 2c). We iteratively coarsen and blur the graph four times by a factor of 2 and produce the expression of every gene at each scale.

Mathematically, each coarsening step produces a pooled version of the graph ${\{{G}^{s}\}}_{s=2}^{5}$ and a coarsening operator ${\{{C}^{s}\}}_{s=2}^{5}$, which is the mapping between nodes at one scale to nodes at the next and allows us to generate pooled versions of the gene expression signals:

$${X}^{s+1},{Y}^{s+1}={C}^{s}{X}^{s},{C}^{s}{Y}^{s}$$

After MS-SSIM, we compute the MSSI between the expression profiles at each scale and return their weighted geometric mean. In detail, we compute the *l*, *c* and *s* SSIM-related values at each scale and derive MSSI based on their weighted product, as for the MS-SSIM:

$${\mathrm{MSSI}}(X,Y,D)={l}_{5}{({X\,}^{5},{Y\,}^{5})}^{{\alpha }_{5}}\mathop{\prod }\limits_{s=1}^{4}c{({X}^{s},{Y\,}^{s})}^{{\alpha }_{s}}s{({X\,}^{s},{Y\,}^{s})}^{{\alpha }_{s}},$$

where the weights are equivalent to those in MS-SSIM^45^:

$$\alpha =(0.0448,\,0.2856,\,0.3001,\,0.2363,\,0.1333)$$

When *X*^*i*^*,Y*^*i*^ are anti-correlated (${\sigma }_{xy} < 0$), *s* is negative, which prevents computing the weighted geometric mean; we, thus, clip negative values to 0. This implies that if, at any scale, *X*^*s*^,*Y*^*s*^ are anti-correlated, the MSSI will be 0, its lowest possible value. We also normalize the original-scale gene expression to be between 0 and 1 but do not re-normalize at each coarsening scale.

### Spatial covariance representation

Our spatial covariance framework includes three components: the COVET statistic, a similarity metric and an algorithm to robustly and efficiently compute the COVET metric. The COVET framework assumes that the interplay between the cell and its environment creates covarying patterns of expression between the cell and its niche, which can be formulated via the gene–gene covariance matrix of niche cells. The COVET statistic constructs a shifted covariance matrix (which preserves algebraic properties of the covariance matrix) and, thus, enables the use of any measure of statistical divergence between covariances to define a principled quantitative similarity metric to compare niches. The key is to build the COVET statistic in such a manner that two COVET matrices are comparable and to design a computationally efficient algorithm to quantify the statistical divergence between them.

#### COVET

The inputs to COVET are (1) the gene expression matrices ($$X\in {R}^{n\times g}$$), where *n* is the number of cells and *g* is the number of genes profiled; (2) the location of each cell in situ; and (3) a parameter (*k*) that defines the number of nearest cells to be included in the niche. For each cell, we identify its *k* nearest cells (excluding the cell itself) based on their spatial proximity and construct a niche matrix $${E}_{i}=\left\{{Y}_{ij}\in\right.$$$$\left.{R}^{g}|\,j\in kNN(i)\right\}$$, which represents the gene expression vector for each of those nearest neighbors. This produces an *n* × *k* × *g* tensor $$\varOmega =\,\left\{{E}_{i}\in\right.$$$$\left.{R}^{k\times g}|i=1,\ldots ,n\right\}$$, which combines the niche matrices of every cell.

The fundamental goal of COVET is to transform those niche matrices into effective representations of a cell’s niche. To this end, we calculate the ‘shifted’ gene–gene covariance matrix between cells in each niche matrix, where, instead of using the classical formulation

$${{\Sigma }_{i}}^{\mathrm{classic}}={\mathrm{Cov}}({E}_{i})=\frac{1}{k}({E}_{i}-{\overline{E}}_{i})^{\mathrm{T}}({E}_{i}-{\overline{E}}_{i})$$

we swap the niche mean expression ${{\overline{E}}_{i}}$ with the total expression average ${\overline{X}}$ (computing the mean over the entire dataset). This enables direct comparison between covariance matrices, as they are constructed relative to the same reference:

$${\Sigma }_{i}={\mathrm{ShiftCov}}({E}_{i})=\frac{1}{k}({E}_{i}-{\overline{{X}}})^{\mathrm{T}}({E}_{i}-{\overline{{X}}}).$$

This creates a representation relative to the entire population, which can better highlight the features that are unique to each niche while also holding the same algebraic properties that the standard covariance matrix holds, namely being positive semi-definite (PSD). Therefore, we can harness measures of statistical divergence to derive a metric on the COVET matrices and quantify differences and similarities between niches. Although we can conceptually use any statistical divergence measure, metrics such as Kullback–Leibler (KL) divergence and Bhattacharyya^30^ distance are too computationally intensive and lack interpretability.

#### Distance between COVET matrices

To meaningfully compare between niches, we cannot simply use the sum difference between two niche matrices *E*_*i*_ and *E*_*j*_, as changing the cells’ order would change the result (whereas there is no meaning to any given order). An intuitive way to quantify niche similarity is by finding the best matching of cells between niche matrices by solving the assignment problem^81^. Optimal transport (OT)^82^ is a relaxed version of the assignment problem, where, instead of matching cells one to one, OT finds the best ‘soft assignment’ between cells. However, because this approach has no closed-form solution and does not scale to large datasets, we can use the closed-form solution of OT between covariance matrices, known as the Fréchet distance^29^, instead:

$${\Delta }_{\mathrm{Fr\acute{e}chet}}({E}_{i},{E}_{j})\,={\mathrm{Tr}}({\Sigma }_{i})+{\mathrm{Tr}}({\Sigma }_{j})-2\cdot {\mathrm{Tr}}(\sqrt{{\Sigma }_{i}{\Sigma }_{j}}).$$

The Fréchet distance has time complexity of *O*(*k*^3^) and is, thus, computationally intractable for large-scale datasets, which would require billions of pairwise computations between all niches. To speed up computation, we swap the matrix square root (MSQR) and product operation in the last term of the Fréchet distance and define the AOT distance as:

$${\Delta }_{\mathrm{AOT}}({E}_{i},{E}_{j})\,={\mathrm{Tr}}({\Sigma }_{i})+{\mathrm{Tr}}({\Sigma }_{j})-2\cdot {\mathrm{Tr}}\left(\sqrt{{\Sigma }_{i}}\sqrt{{\Sigma }_{j}}\right)\,$$

If Σ_*i*_ and Σ_*j*_ are commutative, this is no longer an approximation and Δ_AOT_ = Δ_Fréchet_. Both the approximate and true Fréchet distance require *O*(*k*^3^) operations between each pair of niches and *O*(*n*^2^ *k*^3^) to compute the full distance matrix; however, using the identity that for symmetric matrices, ${\mathrm{Tr}}(AB)=\,\sum _{\gamma ,\delta }{A}_{\gamma \delta }\cdot {B}_{\gamma \delta }$, we arrive at:

$$\begin{array}{c}{\Delta }_{\mathrm{AOT}}({E}_{i},{E}_{j})=\displaystyle\sum _{\gamma ,\delta }\left(\sqrt{{\Sigma }_{{i}_{\gamma \delta }}}\cdot \sqrt{{\Sigma }_{{i}_{\gamma \delta }}}+\sqrt{{\Sigma }_{{j}_{\gamma \delta }}}\cdot \sqrt{{\Sigma }_{{j}_{\gamma \delta }}}-2\cdot \sqrt{{\Sigma }_{{i}_{\gamma \delta }}}\cdot \sqrt{{\Sigma }_{{j}_{\gamma \delta }}}\right)\\ =\displaystyle\sum _{\gamma ,\delta }{\left(\sqrt{{\Sigma }_{{i}_{\gamma \delta }}}-\sqrt{{\Sigma }_{{j}_{\gamma \delta }}}\right)}^{2}={||\sqrt{{\Sigma }_{i}}-\sqrt{{\Sigma }_{j}}\,||}{\,}_{2}^{2}\end{array}$$

Therefore, when working in square root space, we do not require any computationally extraneous matrix multiplication and many calculations of MSQR. Instead, we first calculate the MSQR of each COVET matrix, which is $O(n{k}^{3}$, and then simply calculate pairwise (squared) Euclidean distance for a total time complexity of $O(n{k}^{3}+{n}^{2}{k}^{2})$, which is substantially more efficient than $O({n}^{2}{k}^{3})$ for large *n*. For a given PSD matrix *A*, there could be many possible solutions *B* that fulfill the equation *B*^2^ = *A*. Although this underdetermination is problematic, there is a unique symmetric PSD solution for the MSQR^83^. This solution can be found via spectral decomposition and reconstructing with standard square root of the matrix eigenvalues:

$$\sqrt{A}=\sum _{i}\sqrt{{\lambda }_{i}}{v}_{i}{v}_{i}^{\mathrm{T}},$$

where *λ*_*i*_, *v*_*i*_ are the eigenvalues/vector of *A.*

Because AOT can be formalized as the ${L}_{2}^{2}$ between MSQR of COVET matrices, it allows for direct use of any algorithm that is based on the squared Euclidean distance, such as UMAP, tSNE^84^ and FDL^58^, clustering^31^ and DC^32^ analysis. We can simply compute MSQR of the COVET matrices, flatten the resulting matrices into one-dimensional (1D) vectors and apply the default implementations of all the mentioned algorithms. We can further leverage the squared Euclidean distance representation of the AOT metric and use computational accelerators designed to compute classical pairwise distances for additional speed gains.

We demonstrate that AOT is a good approximation by benchmarking against the true Fréchet distance and the Bhattacharyya distance, another common metric for distances between covariance matrices. Across various sizes of random sets of 64 × 64 covariance matrices, we test the runtime to compute the 10 nearest neighbors matrix in covariance space. As covariance matrices are PSD, to randomly generate *n* covariance matrices of 64 × 64 elements, we first sample *n* random 64 × 64 matrices (using the standard normal) and multiply each by its transpose, as a matrix Gramian is always PSD:

$${{\Sigma }_{i}}_{i=1}^{n}=\left\{{X}_{i}\cdot {X}_{i}^{\mathrm{T}}\right\}_{i=1}^{n};{X}_{i} \sim N(0,{I}_{6{4}^{2}{{\times }}6{4}^{2}}).$$

We find that, whereas AOT produces accurate similarities, its runtime is at least an order of magnitude smaller than that of other metrics, with Fréchet and Bhattacharyya failing on sample sizes larger than 3,000 matrices due to out-of-memory error. Using a GPU implementation of kNN distance built for the Euclidean metric, which can be easily adapted for AOT, the spatial covariance metric is indeed scalable to massive datasets, taking less than 1 min to compute the kNN matrix between 100,000 samples (Extended Data Fig. 1a).

We observe accurate approximation on real COVET matrices, calculated from the eight nearest neighbors of the pharyngeal mesoderm cells from the seqFISH assay^37^, using the 64 most variable genes among the 350 imaged. Despite its efficiency, AOT does not sacrifice accuracy and concurs highly with Fréchet. We calculate the pairwise distance between the pharyngeal mesoderm COVET matrices according to Fréchet, AOT, Bhattacharyya and naive *L*_2_ between matrices. For each pharyngeal mesoderm cell, we find its *k* nearest neighbors for every metric and compute their Jaccard index with the Fréchet nearest neighbors. Across a wide range of *k*, AOT-based kNN is highly congruent with Fréchet kNN, whereas Bhattacharyya and naive *L*_2_ distances are not (Extended Data Fig. 1b). Qualitatively, using Fréchet, AOT and Bhattacharyya pairwise distances to compute two-dimensional (2D) embeddings and PhenoGraph clusters for the COVET matrices returned similar results (Extended Data Fig. 1c,d).

#### Choice of *k*

By default, we select *k* = 8 neighbors to construct COVET, which usually captures the immediate niche of a cell, but the exact choice of *k* should reflect the data. For all datasets analyzed in this study, we kept the value of *k* at the default, demonstrating how finding the optimal *k* is not required to gain insights from ENVI and COVET. Still, given the computational efficiency of both algorithms, we recommend that users attempt a range of *k* values at different scales, such as 8, 20 and 50. Users can visualize the ENVI-learned COVET representations with AOT and choose the most appropriate scale for their biological question. We also implemented an option for COVET to be computed on all cells within a given radius, rather than constant number of neighbors, to account for differences in cell density within a tissue.

### ENVI algorithm

The ENVI algorithm integrates scRNA-seq and spatial transcriptomics data into a common latent embedding, in a manner that can infer spatial context for scRNA-seq and missing genes for spatial data. The core assumption of ENVI is that the interplay between a cell’s phenotype and its microenvironment, as captured by the COVET matrix, empowers better data integration.

ENVI is grounded on autoencoder variational inference but diverges from previous work^9,25,47^. Although current methods only model the expression of genes included in both single-cell and spatial datasets, ENVI explicitly incorporates both microenvironment context for spatial data and expression of the full transcriptome for scRNA-seq data. In addition, ENVI contains two decoders: one for expression, which includes additional neurons that learn gene expression only from scRNA-seq data, and one to predict spatial context. Using these decoders, ENVI trains the VAE^27^ to reconstruct both full transcriptome expression and spatial context from partial transcriptome samples.

To integrate scRNA-seq and spatial data, ENVI learns a common latent space for both data modalities by marginalizing the technology-specific effect on expression via a CVAE^28^. It achieves this by augmenting the standard VAE with an auxiliary binary neuron in the input layers to the encoding and decoding networks representing each data modality. Integration is crucial, as each modality harbors technology-specific artifacts (Extended Data Fig. 2a). ENVI takes as input the scRNA-seq count matrix *X*_sc_ with *n*_sc_ cells and their full transcriptome of *g*_sc_ genes as well as counts of segmented cells from spatial transcriptomics matrix *X*_st_ from *n*_st_ cells and *g*_st_ imaged genes. The algorithm is agnostic to the method used to segment cells before input. It uses the spatial data to compute the COVET matrix for each cell and their MSQR to align with the AOT distance formulation.

Next, ENVI’s conditional autoencoder builds a shared latent space for both data modalities. As the combined embedding must incorporate spatial context and full transcriptome information and must remove confounders relating to modality, we set the latent dimension to 512, substantially larger than standard VAEs in single-cell genomics, which usually contain around 10 neurons^25,34,35^. As input to the encoder, ENVI takes either spatial or scRNA-seq samples (the latter reduced to the subset of genes that have been imaged), along with the auxiliary neuron *c* having value 0 for the spatial data and 1 for scRNA-seq. The expression profile along with the auxiliary neuron are transformed into the latent variable *l* using the same encoding neural network, regardless of data modality:

$$l=\left\{\begin{array}{cc}{\mathrm{Enc}}({x}_{\mathrm{st}},c=0) & {x}_{\mathrm{st}}\in {X}_{\mathrm{st}}\\ {\mathrm{Enc}}({\overline{x}}_{\mathrm{sc}},c=1) & {\overline{x}}_{\mathrm{sc}}\in {X}_{\mathrm{sc}}[:,\,{g}_{\mathrm{st}}]\end{array}\right.,$$

where the encoder returns two vectors, *μ*_*l*_ and *σ*_*l*_, which parameterize a Gaussian with diagonal covariance describing the posterior distribution of the latent. To calculate gradients through random samples, we use the reparameterization trick, which involves generating a sample from the standard normal *ε* ~ *N*(0,1) and describing the latent through a function of *ε*, *μ*_*l*_ and *σ*_*l*_ and treating *ε* as a constant:

$$l \sim N({\mu }_{l},{\sigma }_{l})\,\Rightarrow l={\mu }_{l}+{\varepsilon }\cdot {\sigma }_{l},\,{\varepsilon} \sim N(0,1).$$

Through the training process, our goal is to have the latent encode not only gene expression but also information about the spatial context of a given cell while removing confounding effects to allow transfer learning between modalities. This is achieved by optimizing a single latent space to accurately decode both the full transcriptome and COVET matrix for both data modalities, each missing one of these components. The requisite that the latent space be capable of decoding the spatial niche imbues sufficient spatial information into the latent space during training.

The latent of either modality, along with the appropriate auxiliary neurons, is fed into the ‘expression’ decoder network Dec_Exp_. The loss function, calculated by comparing the activations in the output layer to the true expression profiles, needs to reflect the underlying distribution of each data modality. We use the negative binomial distribution to model scRNA-seq data, similarly to previous work^25,36^, as it suffers from overdispersion and dropout. During training, the scRNA-seq data provide transcriptome-wide expression; therefore, we can include genes whose expression was not provided to the encoder in the loss function, allowing our encoder to model genome-wide expression.

The negative binomial has two parameters per gene: the number of failures, *r*, and success probability, *p*. Thus, the output layer of the decoder consists of $2\cdot {g}_{\mathrm{sc}}$ neurons, where the first *g*_sc_ neurons are the parameter *r* and the latter *g*_sc_ neurons are *p*, using the ‘softplus’ nonlinearity for *r* and the sigmoid function for *p* to keep it a valid probability:

$$p({\hat{x}}_{\mathrm{sc}}=k|NB(r,p))=\frac{k+r-1}{r-1}{(1-p)}^{k}{p}^{r}$$

where

$$r,p={\mathrm{Dec}}_{\mathrm{Exp}}(l,c=1)[:,:\,{g}_{\mathrm{sc}}],{\mathrm{Dec}}_{\mathrm{Exp}}(l,c=1)[:,\,{g}_{\mathrm{sc}}:2{g}_{\mathrm{sc}}]$$

We use the Poisson distribution to model FISH-based multiplexed imaging data due to its high molecular capture rate^3^ and have the first *g*_st_ neurons in the output layer parameterize the per-gene rate parameter *λ* using ‘softplus’ nonlinearity to ensure that it is a valid rate value:

$$P({\hat{x}}_{\mathrm{st}}=k|{\mathrm{Pois}}(\lambda ))=\frac{{\lambda }^{k}{e}^{-\lambda }}{k!}$$

where

$$\lambda ={\mathrm{Dec}}_{\mathrm{Exp}}(l,c=0)[:,:{g}_{\mathrm{st}}]$$

A standard CVAE, in which all neural parameters are shared aside from the auxiliary neurons, is sufficient to simply integrate between scRNA-seq batches, as demonstrated in scArches^34^. However, to successfully integrate scRNA-seq and multiplexed FISH-based technologies, a single auxiliary neuron is not sufficient to regress out all biases. In ENVI, only the first *g*_st_ neurons of the output layer are shared by the two data modalities, whereas the rest are solely trained on the scRNA-seq data. These additional technology-specific parameters improve the ability of ENVI to regress out confounders from the latent embedding, beyond the auxiliary neuron.

Finally, ENVI includes an additional ‘environment’ decoder network Dec_Env_ whose role is to reconstruct the COVET from the latent, which can be trained from the spatial data. The output layer of the environment decoder has $\frac{{g}_{\mathrm{spatial}}\cdot ({g}_{\mathrm{spatial}}+1)}{2}$ output neurons parameterizing the lower triangular Cholesky factor. The Gramian matrix of the output layer is the mean parameter of a standard normal, reflecting our AOT distance, as the log likelihood of the standard normal is the ${L}_{2}^{2}$ distance.

$$P\left({\hat{\Sigma }}^{\frac{1}{2}}={\Sigma }^{\frac{1}{2}}|N(L\cdot {L}^{\mathrm{T}},I\,)\right)=\left(2{\uppi }^{-\frac{{g}_{\mathrm{spatial}}^{2}}{2}}\right)\cdot {\mathrm{e}}^{-\frac{1}{2}{\Vert {\Sigma }^{\frac{1}{2}}}-L\cdot {L}^{\mathrm{T}}\Vert _{2}^{2}},$$

where $$L={\mathrm{Dec}}_{\mathrm{Env}}(l)$$.

The output of the environment decoder is the MSQR of the COVET matrix, which is trained to minimize the ${L}_{2}^{2}$ error with the MSQR of the true COVET matrix. Using the AOT metric in this manner involves computing the MSQR of the COVET samples during training, which can be computationally prohibitive. Instead, we first calculate the MSQR of all COVET matrices, which ENVI is directly trained to reconstruct.

We train ENVI simultaneously on samples from both spatial and single-cell datasets, using mini-batch gradient descent on the variational inference loss. With the learned ENVI model, we impute missing genes for the spatial data by treating the latent embedding of the spatial data as if it were from the single-cell data, using the single-cell auxiliary variable and parameterizing as a negative binomial instead of a Poisson. Conversely, we reconstruct spatial context for the single-cell data by applying the ‘environment’ decoder on its latent, as if it was the latent of the spatial data.

In more detail, we train ENVI to optimize the evidence lower bound (ELBO) with a standard normal prior on the latent, with the goal of increasing the likelihood of the observed data $\{{X}_{\mathrm{sc}},\,{X}_{\mathrm{st}},{\varSigma }_{\mathrm{st}}\}$ for the parameterization of their decoded distributions $\{{\hat{X}}_{\mathrm{sc}},\,{\hat{X}}_{\mathrm{st}},{\hat{\varSigma }}_{\mathrm{st}}\}$ while minimizing the KL divergence between the latent distribution and $N(0,1)$:

$$\begin{array}{l}L={\mathrm{ln}}\,NB({X}_{\mathrm{sc}}|r,p)+{\mathrm{ln}}\,{\mathrm{Pois}}({X}_{\mathrm{st}}|\lambda )+{\mathrm{ln}}\,N({\Sigma }_{\mathrm{st}}|{\mu }_{\mathrm{Env}},I\,)\\\quad-\beta {D}_{\mathrm{KL}}(N\{\,{\mu }_{l},{\sigma }_{l}\},N\{0,1\}\,)\end{array}$$

To train ENVI to impute missing genes for the spatial data, we generate the latent embedding *l*_st_ by passing *X*_st_ through the encoder, and run the latent layer through the ‘expression’ decoder, but with the inverse auxiliary neuron, as if the embedding came from scRNA-seq data:

$${X\,}_{\mathrm{st}}^{\mathrm{Imp}}=E[NB({r}_{\mathrm{st}},{p}_{\mathrm{st}})],$$

where

$$r,p={\mathrm{Dec}}_{\mathrm{Exp}}({l}_{\mathrm{st}},c=\,1)[:,:{g}_{\mathrm{sc}}],{\mathrm{Dec}}_{\mathrm{Exp}}({l}_{\mathrm{st}},c=1)[:,{g}_{\mathrm{sc}}:2{g}_{\mathrm{sc}}].$$

Similarly, we reconstruct the spatial context for dissociated scRNA-seq samples by passing the scRNA-seq latent embedding *l*_sc_ through the ‘environment’ decoder:

$${X}_{\mathrm{sc}}^{\mathrm{Env}}=E\left[N({\mu }_{\mathrm{sc}}^{\mathrm{Env}},1)\right]\,{\rm{where}}\,{\mu }_{\mathrm{sc}}^{\mathrm{Env}}={L}_{\mathrm{sc}}\cdot {L}_{\mathrm{sc}}^{\mathrm{T}},{L}_{\mathrm{sc}}={\mathrm{Dec}}_{\mathrm{Env}}({l}_{\mathrm{sc}}).$$

To allow flexibility in modeling technologies with different count distributions and molecular capture rates, we implemented the normal, Poisson, negative binomial and zero-inflated negative binomial (ZINB)^85^ distributions, which can be chosen for either modality to reflect pre-processing steps or varying levels of noise or dropout. The rate or mean parameters (*λ* for Poisson, *r* for NB and ZINB and *μ* for normal) must all be defined per cell and per gene and shared across the single-cell and spatial data. However, all other parameters can be chosen to be either per cell and per gene or simply per gene and can be either shared between technologies or made distinct.

By default, the encoder and two decoder networks consist of three hidden layers, each with 1,024 neurons. The latent embedding consists of 512 neurons, and the prior coefficient is set to *β* = 0.3. For small datasets whose total samples size is fewer than 10,000 cells, we recommend increasing the reliance on the prior and set *β* = 1.0. We train ENVI for two^14^ gradient descent steps with the ADAM optimizer^86^ with learning rate 10^−3^ (lowered to 10^−4^ during the last quarter of training steps) and a batch consisting of 1,024 samples, half taken from scRNA-seq and the other half taken from spatial data. To reduce computational complexity, we subset the scRNA-seq dataset to the union of the 2,048 highly variable genes and all genes included in the spatial dataset rather than the full transcriptome.

ENVI training is constant in both time and memory, whereas methods such as Tangram and NovoSpaRc scale quadratically with dataset size and cannot be GPU accelerated on datasets above a few thousand cells. We benchmarked the run times of ENVI, Tangram^9^, NovoSpaRc^26^, gimVI^25^, uniPort^47^, deepCOLOR^48^ and Harmony^49^ on scRNA-seq datasets of various sizes and on osmFISH, seqFISH, Xenium and MERFISH datasets. All models were trained with their default parameters using a single 12 GB GeForce RTX 2080 GPU, except Tangram, which produced an out-of-memory error above 10,000 cells and was trained with a CPU instead. Model training was stopped prematurely if it exceeded 5 h.

As expected, ENVI’s training time was consistently around 10 min regardless of dataset size (Extended Data Fig. 2b), and Harmony was also constant in time. gimVI runtime grew linearly with dataset size (the model trained for a predefined number of epochs over the datasets), and NovoSpaRc and Tangram were prohibitively slow on larger spatial and scRNA-seq datasets (they learn a cell-to-cell mapping between the spatial and single-cell datasets). We found that GPU acceleration is not possible for Tangram. deepCOLOR and uniPort were also substantially slower than ENVI at larger cell numbers.

### Evaluation of integration quality

Batch average silhouette width (bASW), introduced in a recent benchmarking of batch integration methods for scRNA-seq atlases^41^, evaluates latent integration based on mixing between batches and co-localization of similar cell types within the latent. In brief, bASW computes, for each cell type, how well-mixed batch labels are using the silhouette coefficient and returns the average across all cell types. By treating each modality as a different batch, we could use the bASW score to measure the quality of ENVI’s learned latent. The latent of ENVI is large, consisting of 512 neurons; because silhouette coefficient is affected by the curse of dimensionality, we first compressed the ENVI latent to the top 10 principal components and computed bASW on them.

### Benchmarking imputation

We benchmarked ENVI gene imputation following previous approaches^25,44^ that generate a test set of held-out genes using cross-validation and compared imputed and true expression using Pearson correlation and our spatially aware MSSI metric. We evaluated log expression and imputation profiles, with pseudocount 0.1.

Many algorithms use scRNA-seq data to impute missing genes in spatial transcriptomics data^42,43,87–89^. We compared ENVI to gimVI, Tangram and uniPort for their competitive performance^44,47^, NovoSpaRc for its use of spatial context and optimal transport for data integration, deepCOLOR^48^ for its use of a deep generative model and Harmony^49^ for its prevalence as a batch correction method^50^.

On the osmFISH dataset, which includes only 33 genes, we performed a full leave-one-out cross-validation by hiding every gene in the imaging panel individually and predicting its expression. On the seqFISH, MERFISH and Xenium datasets, which assay hundreds of genes, we used five-fold cross-validation, whereby the imaged gene set was divided into five random groups, and each model was tested on one withheld group after training on four. To appraise performance, we used a ‘relative’ one-sided *t*-test, as scores are paired across genes.

We benchmarked all models using their default parameters and instructions on all datasets:

- gimVI: We trained for 200 epochs with a batch size of 128 and latent dimension, per author recommendations (https://docs.scvi-tools.org/en/stable/), and parameterized spatial and scRNA-seq datasets with NB and ZINB distributions, respectively. To impute genes with the trained model, we followed manuscript instructions and trained a kNN regression model on the scRNA-seq latent and full transcriptome expression, setting *k* as 5% of cells in the single-cell dataset. We then applied the regression model on the spatial data latent to predict the expression of unimaged genes.
- Tangram: We trained for 1,000 epochs using default parameters (https://github.com/broadinstitute/Tangram). For osmFISH, seqFISH and Xenium datasets, we used the default ‘cells’ mode, and, for the much larger MERFISH atlas, we used the ‘cell-type’ mode, per the tutorial. We set the density prior to be uniform, as our spatial benchmark datasets are single-cell resolution. With the learned mapping, we used the ‘project_genes’ function to impute genes from scRNA-seq onto the spatial dataset.
- NovoSpaRc: We followed the repository instructions (https://github.com/rajewsky-lab/novosparc), using an ‘alpha’ coefficient on a spatial location prior of 0.25 and smoothness parameter ‘epsilon’ of 0.005. To compute the scRNA-seq pairwise distance matrix, we used the union of the 2,048 most variable genes and all genes in the spatial dataset. For spatial datasets consisting of multiple samples, we trained a different model on each sample. Because NovoSpaRc does not scale well to large datasets, we reduced the MERFISH-related scRNA-seq dataset to a tenth of its size, sampling uniformly across each cell type. We applied the learned mapping to impute missing genes using the ‘tissue.sdge’ function.
- uniPort: We replicated tutorial instructions for integrating spatial and single-cell datasets (https://uniport.readthedocs.io/) by normalizing each dataset according to library size, log transforming counts, executing the ‘batch_scale’ function, training the model for 30,000 iterations with a ‘lambda_kl’ value of 5.0 and, finally, predicting the expression of hidden genes using the ‘predict’ function.
- deepCOLOR: We trained for 500 epochs using default parameters from the tutorial (https://github.com/kojikoji/deepcolor). deepCOLOR does not directly impute unimaged genes, so we multiplied the resulting mapping matrix with the scRNA-seq expression of the hidden genes to predict their expression.
- Harmony: We treated spatial and single-cell datasets as separate batches and integrated them using the default Harmony implementation in scanpy^90^ (https://scanpy.readthedocs.io/). We only included genes from the scRNA-seq data that were also in the spatial data (and removed test genes) to produce Harmony embeddings from the principal components of the concatenated dataset. Mirroring gimVI’s imputation procedure, we performed kNN regression on the Harmony embeddings to reconstruct expression of the manually hidden genes.

### Impact of data sparsity on ENVI

To validate ENVI’s robustness to single-cell or count data sparsity, which can affect integration^9^, we benchmarked the full embryogenesis seqFISH and scRNA-seq data against random subsampling to 90% or 80% of counts according to the binomial distribution. For all three datasets, we performed five-fold cross-validation (see the ‘Benchmarking imputation’ subsection for details), finding that removing even 20% of counts does not greatly impact ENVI performance, which still surpasses Tangram on the full dataset (Extended Data Fig. 2c). Using a kNN (*k* = 5) classifier trained to predict cell type from the scRNA-seq latent space, we assigned labels to the seqFISH data and measured balanced accuracy compared to the original assignment, finding that the ENVI latent space remains reliable upon downsampling; datasets with higher sparsity are only slightly less accurate (Extended Data Fig. 2d).

### FDL and DCs

FDL^58^ and DCs^32^ capture and visualize continuous trends in single-cell data^19^. We calculated FDL following the implementation in Van Dijk et al.^91^, by computing a kNN matrix (using default *k* = 30), converting to an affinity matrix using an adaptive Gaussian kernel with width = 30 and *k* *=* 10, symmetrizing the matrix and using the ForceAtlas^92^ function ‘force_directed_layout’ for visualization. DC computation followed a similar process to compute a data affinity matrix, except that we multiplied the affinity matrix by the inverse of its degree matrix to compute the normalized Laplacian. The eigenvectors of the Laplacian matrix, in order of eigenvalue magnitude, are the DCs.

### Applying ENVI to seqFISH embryogenesis data

We started with pre-processed data from the E8.75 mouse gastrulation study^37^, which included 351 genes measured with seqFISH (57,536 imaged cells), and paired it with E8.5 scRNA-seq data (12,995 cells) from a second study^38^. We further processed the scRNA-seq data by removing mitochondrial genes, genes expressed in less than 1% of cells, cells with library size greater than 33,000 (set manually to match the knee point) and cells annotated as ‘nan’ or representing doublets. To avoid confounding batch effects^50^, we used only the largest scRNA-seq batch (labeled ‘3’). For the seqFISH dataset, we used only the first of three imaged embryos (‘embryo1’), removed cells with abnormally high total expression (threshold set manually to 600) and removed the gene *Cavin3*, which did not appear in the scRNA-seq dataset. For both datasets, we used cell type annotations provided by the authors and visualized the seqFISH data using spatial coordinates and the scRNA-seq data using a UMAP embedding (Fig. 2a). We also renamed several cell types to resolve nomenclature differences, including changing presomitic mesoderm to somitic mesoderm and splanchnic mesoderm to pharyngeal mesoderm.

We trained ENVI on the union of the 2,048 most variable genes in the scRNA-seq data, all seqFISH-measured genes, all *HOX* genes and several organ markers (Supplementary Table 1) using default parameters. We visualized the learned latent posterior of the seqFISH and scRNA-seq datasets using UMAP and found that cell types tend to co-embed regardless of modality (Fig. 2b).

To test the imputation of unimaged canonical organ markers *Ripply3* (ref. ^51^) (lung), *Nkx2-5* (ref. ^52^) (heart) and intestine *Tlx2* (ref. ^53^) (intestine), we visualized their imputed z-scored, logged expression and thresholded values lower than 2, finding almost exclusive expression in the correct organ (Fig. 2f). To confirm that expression in the correct location at E8.75, before organ formation, we imaged each marker gene using whole-mount HCR (Fig. 2f; see the ‘Whole-mount HCR’ subsection). HCR produces per-gene three-dimensional (3D) images, which we oriented coronally to match the seqFISH data. We similarly trained gimVI and Tangram on the complete scRNA-seq and seqFISH datasets to impute *Ripply3*, *Nkx2-5* and *Tlx2* and visualized as for ENVI imputation, finding that ENVI imputation more closely matches the experimental data (Extended Data Fig. 3b).

### Spatial organization of emerging organs

At E8.5, scRNA-seq cell clusters correspond to primordial endodermal organs, ordered by where they will later emerge along the gut tube^55^. We identified organ-specific gene sets (Supplementary Table 2) by using the ‘rank_genes’ function in scanpy^91^ to apply a Wilcoxon test for differentially expressed genes in each organ in a reference scRNA-seq endodermal atlas^55^. Thymus and thyroid are not well delineated at this stage, so we collapsed them into a single thymus/thyroid label, and we assigned small intestine and colon cells to a single ‘intestine’ label to avoid inconsistencies, as the seqFISH tissue section does not include colon^37^.

We used PhenoGraph to cluster the scRNA-seq gut tube cells into 12 clusters and labeled clusters by best matching organ based on z-scored and logged expression of each gene set, averaged across all cells in that cluster. Most clusters are highly distinct, whereas some co-express several programs. We labeled clusters for which the (z-scored) ratio between the highest and second-highest expressed gene set is above 1.5 with the most highly expressed organ. To assign ambiguous clusters with ratios below 1.5, we inspected marker expression individually:

- Cluster5: Thymus/thyroid gene set expression is highest, but because lung marker *Ripply3* (ref. ^51^) and *Irx1* (ref. ^93^) expression is high (average z-score logged expression, 0.90) while thymus/thyroid marker *Nkx2-1* (refs. ^52,94^) is low (−0.15), we labeled Cluster5 as ‘dorsal lung’ (second-highest expressing organ).
- Cluster6: Dorsal lung gene set expression is highest, with pancreas a close second. Because the cluster has minimal *Ripply3* and *Irx1* expression (0.18) but is enriched for pancreas marker *Pdx1* (ref. ^95^) expression (0.43), we labeled Cluster6 cells as pancreas.
- Cluster7: Pancreas and liver gene set expression is highest and second highest, respectively. Due to high *Pdx1* expression (0.99) and low liver marker *Ppy*^96^ expression (−0.12), we kept the pancreas label for this cluster.

We inferred COVET representations for the scRNA-seq gut tube cells using the trained ENVI model and then measured pairwise AOT distances between the conjoined set of seqFISH and scRNA-seq COVET matrices to generate UMAP embeddings and PhenoGraph clusters. The data generated seven COVET clusters (CC0–CC7), which are highly congruent with emerging organs in the scRNA-seq data, indicating their spatial delineation (Extended Data Fig. 4a): thymus/thyroid cells were assigned to CC0 (75%) or the spatially proximal CC1 (17%); dorsal lung cells were assigned to CC1 (52%) or CC0 (36%); ventral lung cells were assigned to CC2 (62%) or the highly related clusters CC1 (12%) or CC3 (19%); liver cells were assigned to CC2 (94%); pancreas cells were assigned to CC3 (58%) or the related cluster CC2 (26%); and intestine cells were assigned almost entirely to CC4–CC7, with only 1% assigned to CC3.

Gut tube cells in the seqFISH data were assigned organ labels via their COVET representations. We fit an AOT metric kNN classifier (*k* = 5) on the scRNA-seq ENVI COVET matrices and their organ labels and used the classifier to assign budding organ labels to seqFISH COVET (Fig. 3a). Projecting labels back onto their seqFISH coordinates reveals the spatial pattern of organogenesis, from thymus/thyroid to the lung compartments, liver, pancreas and intestine and colon from anterior to posterior (Fig. 3b).

To calculate average COVET matrices predicted by ENVI (scRNA-seq organs) or measured directly (seqFISH), we compute the AOT average for the matrix set by calculating the matrix square of mean of their MSQR:

$$\widehat{\mathrm{COVET}}={\left(\frac{1}{n}\mathop{\sum }\limits_{1}^{n}\sqrt{\mathrm{COVET}_{i}}\right)}^{2}$$

Mean COVET matrices are highly congruent between the two datasets for both dorsal and ventral lung cells, although the scRNA-seq COVET matrices are slightly smoother as they were inferred by ENVI rather than measured (Fig. 3c). To find gene groupings, we performed hierarchal clustering on the 64 genes in each mean COVET matrix, finding that *Dlk1*, *Gata4*, *Gata5*, *Aldh1a2* and *Foxf1* covary in the ventral lung COVET but not in the dorsal lung, whereas *Tagln*, *Six3*, *Thbs1*, *T* and *Epcam1* exhibit the opposite pattern.

We generated clusters of each COVET matrix by plotting their average expression in cells near the anterior gut tube (fewer than 50 distance units away), but not the gut tube itself, and found that ventral niche genes are enriched in the pharyngeal mesoderm, whereas dorsal niche genes localize to brain and cranial mesoderm (Fig. 3d). As pharyngeal mesoderm is ventral to the gut, and brain and cranium are dorsal, the uniquely covarying genes in the COVET matrices allow us to reconstruct each lung compartment’s spatial context.

We also assigned budding organs using integration methods that do not model spatial context (gimVI and Tangram) and computed ENVI without COVET to highlight the importance of explicit modeling of microenvironment:

- gimVI: We trained gimVI on the full embryogenesis scRNA-seq and seqFISH datasets using defaults in the ‘Benchmarking imputation’ subsection (10 latent dimension, 200 epochs, NB for spatial and ZINB for single cell). We took the subset of gut tube cells in each modality from the learned latent embedding of scRNA-seq and seqFISH data and similarly learned a kNN classifier (*k* = 5) from the single-cell latent and organ assignment, using it to predict labels on the spatial latent.
- Tangram: Using parameters in the ‘Benchmarking imputation’ subsection (1,000 epochs, uniform density prior, ‘cells’ mode), we trained Tangram to learn a mapping matrix from scRNA-seq to spatial data. We subset the Tangram matrix to the mapping from scRNA-seq gut tube to seqFISH gut tube cells and re-normalized the columns to sum to 1. We transferred organ labels using Tangram’s ‘ project_cell_annotations’ function, which uses the subsetted mapping matrix to calculate the probability of each organ being assigned to each seqFISH gut tube cell, and we labeled according to the most probable organ.
- ENVI without COVET: We retrained ENVI to solely reconstruct gene expression profiles, excluding any COVET-related information. A kNN classifier (*k* = 5) on the learned latent was used to transfer organ labels from the scRNA-seq gut tube onto seqFISH cells.

Due to the lack of gene vocabulary and small number of gut tube cells, other methods could not assign labels as reliably as ENVI: gimVI failed to delineate dorsal lung from thymus/thyroid cells and missed almost all liver cells, and Tangram’s labeling lacked coherent spatial structure (Extended Data Fig. 4b). Without COVET, ENVI was unable to distinguish between ventral lung and liver, although its results most closely resembled the COVET-based assignment and known organ organization.

### ENVI robustness to neighborhood size

The optimal number of neighbors used to construct COVET depends on dataset features and desired analysis (see the ‘Spatial covariance representation’ subsection), but ENVI is nevertheless robust to variations of this parameter. For the seqFISH dataset, we calculated COVET matrices with *k* = 6, 8, 10 or 12 nearest neighbors (original, *k* = 8) and retrained ENVI on each representation. For each of the four ENVI models, we assigned organ labels onto the seqFISH gut tube cells, again using a kNN classifier on COVET matrices in AOT space. Despite doubling neighborhood size and inherent stochasticity in training deep learning models with batch gradient descent, all versions reliably assigned cells to spatial context (Extended Data Fig. 5a). Although there are some differences, even the worst-performing mode (*k* = 6), which mislabeled many dorsal lung cells as thymus/thyroid, is more accurate than competing methods (Extended Data Fig. 4b).

### AP polarity of developing spine and NMP cells

Spinal cord cells and their NMP precursors in the seqFISH data (total, 2,830 cells) span the embryo AP axis and make up a substantial fraction of cells in the scRNA-seq data (1,289 cells, 10% of total). To gauge whether ENVI can correctly map these cells and spatial trends along the AP axis, we first combined empirical seqFISH and ENVI-inferred scRNA-seq COVET matrices from spine and NMP cells and computed DCs via eigendecomposition of the Laplacian of the AOT kNN (*k* = 30) graph in COVET space. We then compared to DCs of spatial coordinates of seqFISH spine and NMP cells, calculated using a kNN (*k* = 30) graph with standard Euclidean distance, finding that pseudo-AP coordinates based on COVET DC are highly congruent with true AP coordinates based on seqFISH DCs (Fig. 4b and Extended Data Fig. 6a) and (logged) expression of known posterior and anterior genes (Fig. 4c,d).

We attempted to reconstruct pseudo-AP axes for gimVI and Scanorama. For gimVI, we used the model trained on the complete embryogenesis datasets and subset the learned gimVI combined latent to only the spine and NMP cells from the spatial and scRNA-seq datasets. We calculated the top three DCs from the latent embeddings and found that DC 2 was most correlated with true AP polarity (seqFISH spine and NMP cells), *r* = 0.76. Scanorama is designed for batch integration and uses mutual nearest neighbors to directly correct the gene expression count matrix and remove batch effect. Following scanpy instructions (https://scanpy.readthedocs.io/en/stable/), we applied Scanorama to produce integrated count matrices of the seqFISH and scRNA-seq spine and NMP cells. We computed DCs from the combined Scanorama-corrected scRNA-seq and spatial datasets and found that DC 3 is most correlated with true AP, *r* = 0.70. Unlike ENVI, both of these methods produced spine and NMP cells in the posterior with low DC values (Extended Data Fig. 6b). We note that, because DC order is arbitrary, we reversed any DC negatively correlated with the true AP. Tangram was excluded from this analysis as it does not calculate a combined embedding from which we can recover a pseudo-AP axis.

To assess the accuracy of pseudo-AP mapping, we ordered scRNA-seq spinal cells by pseudo-AP value and examined expression of canonical markers *Rfx4* (ref. ^63^) (anterior), *Hoxaas3* (ref. ^61^) (posterior) and *Hoxb7* (ref. ^64^) (posterior) (Fig. 4e). Gene expression values were logged and z-scored, and ordered profiles were smoothed with a first-order Savitzky–Golay filter with window size 128 for visual clarity.

To determine the quality of the pseudo-AP axis predicted for scRNA-seq spinal cells by each method, we calculated its correlation with the logged expression of known posterior genes *Hoxaas3*, *Hoxb5os*^61^, *Hoxb9* (ref. ^60^), *Hoxb7* and *Tlx2* (ref. ^97^) and anterior genes *Foxa3* (refs. ^98,99^), *Hoxd3* (ref. ^59^), *Hoxa2* (refs. ^100–102^), *Rfx4* and *Hoxd4* (Extended Data Fig. 6c), providing a quantitative recapitulation of pseudo-AP-ordered expression (Fig. 4e).

### Inferring *Sst* neuron cortical depth with MERFISH

We used the BICCN’s 252-gene MERFISH primary motor cortex atlas^40^ and its matching scRNA-seq reference^70^ to demonstrate ENVI in a tissue-wide context. For the single-cell data, we removed cells lacking cell type annotations or labeled as doublets or low quality, leaving 71,183 cells across three samples, and removed genes that both (1) appear in less than 5% of cells and (2) are not in the MERFISH panel. For the MERFISH data, we included all 12 samples, for a total of 276,556 cells from 64 motor cortex slices, and we removed cells lacking a cell type label and the genes *Crispld2* and *Igf2*, as they were absent from the scRNA-seq data, but avoided any additional pre-processing. Both spatial and scRNA-seq datasets were labeled into neuronal and non-neuronal cell types. For brevity and consistency between datasets, we relabeled the MERFISH GABAergic neurons from ‘Sst-chodl’ to ‘Sst’ and collapsed the ‘PVM’, ‘macrophage’ and ‘microglia’ labels to ‘microglia’.

We used five-fold cross-validation to benchmark ENVI imputation against Tangram, gimVI, NovoSpaRc, uniPort, Harmony and deepCOLOR with default parameters (see the ‘Benchmarking imputation’ subsection), except that we applied Tangram with ‘cell-type’ mode, which averages single-cell data per cell type, and ran NovoSpaRc independently for each slice, subsampling scRNA-seq data to 10% of each original size, because these methods do not otherwise scale to these data. ENVI MSSI and Pearson correlations were significantly higher than other methods (Extended Data Fig. 7a), and ENVI imputation of unimaged genes matches ISH from the Allen Brain Atlas (Extended Data Fig. 7b).

The full transcriptome information in scRNA-seq data allowed finer subtyping than the 22 cell types in the MERFISH dataset. Specifically, we further divided the *Sst* interneurons into nine subtypes and extracted gene sets for each subtype using the scanpy ‘rank_genes_group’ function. For the subset of MERFISH genes present in each gene set, we calculated average expression in every MERFISH *Sst* cell. We measured the pairwise correlation between gene sets within each modality and found that each subtype was delineated much more specifically in the single-cell data (Fig. 5b). We quantified this by computing the per-gene-set entropy across the pairwise correlation matrix, after normalizing with ‘softmax’. The entropy for each gene set was higher in the MERFISH data, demonstrating the lack of distinction between subtypes.

To map the labeled scRNA-seq *Sst* interneurons to their cortical depth, we embedded ENVI-imputed scRNA-seq COVET matrices and MERFISH COVET matrices into DCs and FDL via a kNN graph (*k* = 100) on AOT distance. The first COVET DC corresponds to pseudodepth and matches the cortical depth of MERFISH cells visualized on a single slice (Fig. 5c) and aligns with the primary axis of the COVET FDL (Fig. 5d). For each scRNA-seq *Sst* neuron, we predicted cortical depth using the pseudodepth axis (COVET DC 1), grouped the results by subtype and plotted their distribution (Fig. 5e).

### osmFISH imaging of somatosensory cortex

We applied ENVI to a 33-gene osmFISH dataset (4,530 cells, one sample) and complementary scRNA-seq dataset (30,005 cells) of the somatosensory cortex^3^, using the authors’ cell type annotations and no additional processing besides removing genes expressed in less than 1% of cells in the scRNA-seq data (Extended Data Fig. 8a). As osmFISH data are more dispersed than MERFISH and seqFISH (Extended Data Fig. 2a), we modeled them with the negative binomial instead of the Poisson distribution. Due to the limited size of the scRNA-seq dataset, we changed its parameterizing distribution from NB to ZINB. Because the total sample size is small (fewer than 10,000 cells), we also increased the reliance on the prior latent distribution and increased the regularization to *β* = 1.0, which is common practice in Bayesian modeling.

We visualized and compared the ENVI and gimVI learned latent spaces with a UMAP embedding labeled by cell type annotations from the osmFISH and scRNA-seq datasets. The ENVI embedding separates distinct cell types, with similar labels from the two data modalities occupying similar spaces (Extended Data Fig. 8b), whereas gimVI confuses oligodendrocytes and pyramidal neurons and cannot accurately co-embed osmFISH and scRNA-seq endothelial cells (Extended Data Fig. 8c).

We quantified integration quality and calculated the average center-of-mass embedding for each cell type, from both seqFISH and MERFISH datasets, in the gimVI and ENVI embedding spaces. ENVI and gimVI latent dimensions are vastly different in size (512 for ENVI compared to only 10 for gimVI), so we normalized each column in the pairwise distance to a maximum value of 1. In the ENVI latent, the center of mass for each osmFISH cell type is distinctly closer to its counterpart in the scRNA-seq data compared to other cell types, whereas cell types are less well separated in the gimVI latent (Extended Data Fig. 8d). For each cell in the scRNA-seq data, we quantified this as the ratio of its five osmFISH nearest neighbors in the latent space that share its cell type and averaged across the six cell types. The latent cell type agreement was 0.58 for ENVI and 0.38 for gimVI.

Using leave-one-out cross-validation, ENVI outperformed alternative methods on spatial imputation (Extended Data Fig. 8e). We further imputed the expression of three unimaged genes onto the osmFISH dataset using the full ENVI model (Extended Data Fig. 8f) and validated by comparing them to Allen Brain Atlas ISH images of the somatosensory cortex. ENVI imputation and ISH images both specify *Dti4l*, *Rprm* and *Ndst* expression in the L2/3, L5–L6 and CA1 regions, respectively. The Allen Brain Atlas provides both raw ISH images and processed, cell-segmented expression profiles. Because each view is difficult to interpret on its own, we overlaid the processed profiles on top of the raw ISH images for clarity.

### Xenium data analysis of LM

We assayed a slice of mouse brain bearing a LM of melanoma using snRNA-seq and Xenium (see the ‘Generation of mouse melanoma LM FFPE-snRNA-seq and Xenium datasets’ subsection). Raw Xenium imaging data were processed using the default pipeline provided by 10x Genomics^16^ to produce a segmented cell-by-gene count matrix. In brief, nuclear segmentation was applied on DAPI stains, and all RNA molecules in each segmented mask and within a 15-μm dilation were assigned to cells to compose a count matrix.

We further filtered the Xenium data by removing cells with library size less than 10 and more than 300 and kept only genes that were in the snRNA-seq data. For the snRNA-seq data, we only kept cells with library size less than 10,000 and removed mitochondrial genes and any gene expressed in less than 5% of cells, unless it was in the Xenium panel. Finally, we removed any doublets predicted by DoubletDetection^103^ from either dataset, followed by median library size normalization on the snRNA-seq data. This process resulted in 243 genes captured in 74,132 cells in the Xenium dataset and 9,230 genes sequenced in 9,870 cells by snRNA-seq.

To assign cells to cell types, we independently clustered each dataset with PhenoGraph and searched for per-cluster marker genes using the scanpy ‘rank_genes_groups’ function. We first labeled Xenium data by neuron, endothelium, oligodendrocyte, tumor, astrocyte and immune/fibroblast groups. We then reclustered neurons and annotated into excitatory and inhibitory compartments according to expression of *Slc17a7* and *Gad1* and separated immune/fibroblast into immune cells and fibroblasts. The snRNA-seq data followed a similar hierarchical process, except that fibroblasts and immune cells were distinguished in the first round of clustering. According to an independently curated set of genes for each group^75^, our cell typing matched known transcriptional markers (Extended Data Fig. 9a). We benchmarked ENVI imputation against competing methods as for other methods (see the ‘Benchmarking imputation’ subsection), finding that ENVI outperforms all methods except for Harmony according to Pearson correlation but does equally well according to MSSI (Extended Data Fig. 9b).

To evaluate cell type label transfer from snRNA-seq to Xenium data for ENVI, gimVI, Harmony, deepCOLOR and uniPort, we fitted a kNN (*k* = 5) classifier on the snRNA-seq latent to predict cell type labels and used it to assign labels to the Xenium data. Tangram does not use a latent, so we used its ‘project_cell_annotations’ function and labeled each Xenium cell according to the most probable snRNA-seq cell type mapped to it. NovoSpaRc does not assign cell type labels and was not compared. We measured transfer accuracy with balanced accuracy, the per-cell-type arithmetic mean of precision and recall, averaged across cell types (Extended Data Fig. 9c). ENVI transferred information as accurately as Tangram, uniPort and Harmony and was only slightly superseded by gimVI, validating our cell type annotation label transfer.

To uncover the relationship between phenotype and environment for each cell type in the Xenium dataset, we measured the agreement between clusters derived from expression (phenotype) and COVET representations (environment). Because non-parametric methods are biased by sample size, for each cell type we performed *k*-means (*k* = 5) clustering on the logged expression of its cells and separately on its COVET representations. Each clustering was performed 10 times with random starting points. For each cell type, pairwise adjusted Rand index (ARI) was computed between each expression and COVET clustering, for a total of 100 values, and we reported their mean (Fig. 6c).

Unlike excitatory neurons, whose localization pattern is mirrored in their transcriptional profiles, the niche of immune cells in the Xenium dataset (canonically either brain-resident microglia or tumor-colonizing macrophages^75^) was not reflected in their gene expression. We attempted to divide the immune cells into macrophages or microglia (Supplementary Table 4) by computing the average logged expression of each cell type marker gene set in PanglaoDB^75^ for every immune cell in the snRNA-seq and Xenium datasets, using only the subset of genes present in the Xenium assay (Fig. 6d). The high degree of overlap between macrophage and microglia genes in the spatial data may explain why, unlike the snRNA-seq data, expression and microenvironment corresponded poorly for immune cells.

We mapped annotated snRNA-seq immune cells to spatial context using the COVET predictions from ENVI. PhenoGraph clustering of snRNA-seq and Xenium immune COVET representations revealed major microenvironment clusters C0, representing immune cells in the cortex surrounded by excitatory neurons, with 80% of snRNA-seq cells annotated as microglia; C1, representing immune cells in the basal ganglia, dominated by inhibitory neuron environments, with 80% of snRNA-seq cells annotated as microglia; and C2, representing cells in and around the tumor, with 90% of snRNA-seq cells annotated as macrophages. These strong associations predict that macrophages are localized to the tumor and its boundary, whereas microglia localize mainly to basal ganglia and cortex, recapitulating the known tendency for brain tumors to recruit bone-marrow-derived macrophages^76,77^.

For further interpretability, ENVI can also invoke the inferred COVET representations and explicitly predict the microenvironment composition of each snRNA-seq cell. For each cell in the Xenium dataset, we counted the instance of each cell type within its *k* = 8 nearest neighbor microenvironment, resulting in a *n*_*Xenium*_ by |*C*| matrix titled *M*, where |*C*| = 8 is the number of distinct cell types. We then fit a kNN (*k* = 5) regression model to predict *M* from COVET representations of the Xenium data. The trained model was applied to the COVET matrices that ENVI predicted for the snRNA-seq data to infer the distribution of cell types in each cell’s niche. As for COVET-based clusters, macrophage niches predicted from the snRNA-seq data were highly enriched for tumor cells, whereas microglia niches contained more inhibitory neurons and oligodendrocytes (Fig. 6g).

ENVI can also be extended to identify markers of different macrophage types. Remsik et al.^75^ identified *Ccr2*, *Ms4a4c* and *Lst1* as infiltrating monocyte markers based on cellular indexing of transcriptomes and epitopes by sequencing (CITE-seq) analysis. ENVI imputation of these genes on Xenium immune cells is indeed specific to cells within the tumor (Fig. 6h).

Despite accurately transferring cell type information and imputing missing genes (Extended Data Fig. 9b,c), the absence of direct spatial modeling prevents gimVI and Harmony from reliably inferring subtype-specific microenvironments. We clustered gimVI embeddings of snRNA-seq and Xenium immune cells and found no obvious tumor-related cluster; 90% of snRNA-seq macrophages and 68% of microglia were assigned to gimVI cluster C1, prohibiting clear assignment of subtype to microenvironment (Extended Data Fig. 9d,e). Similarly, gimVI imputation of tumor infiltration genes did not distinctly enrich for immune cells within the tumor, and, despite outperforming ENVI according to Pearson correlation on imaged genes, Harmony also failed to accurately impute the expression of tumor-infiltrating markers (Extended Data Fig. 10).

### Experimental methods

#### Whole-mount HCR

Whole-mount HCR mRNA in situ was performed as described previously^54^, with minor modifications^104^. Mid-gestation embryos at E8.75 were treated with 10 µg ml^−1^ proteinase K for 5 min at room temperature, followed by washing and post-fixation in 4% paraformaldehyde (PFA) for 20 min. Embryos were incubated in hybridization buffer supplemented with 2 pmol of each probe (*Ripply3*, *Nkx2-5* or *Tlx2*) overnight at 37 °C, followed by an amplification step with 60 pmol of each fluorophore-conjugated hairpin for 12–16 h at room temperature. Embryos were then stained with 0.5 µg ml^−1^ DAPI (Thermo Fisher Scientific) and cleared using a modified Ce3D+ clearing protocol^105^ for 24–48 h. Images were acquired on a Nikon A1R laser scanning confocal microscope with a ×10 objective and 3.0-µm *z*-step size. Image rendering and optical sections were generated using IMARIS (version 9.9.0, BitPlane). All probes, hairpins and buffers were designed by and purchased from Molecular Instruments.

#### Generation of mouse melanoma LM FFPE-snRNA-seq and Xenium datasets

Animal studies were approved by the Memorial Sloan Kettering Cancer Center Institutional Animal Care and Use Committee under protocol 18-01-002. Mice were housed in specific pathogen-free conditions, in an environment with controlled temperature and humidity, on a 12-h light/dark cycle (lights on/off at 6:00/18:00), and with access to regular chow and sterilized tap water ad libitum. For this study, an 8-week-old female C57Bl/6-*Tyr*^c-2^ mouse (The Jackson Laboratory, 000058, albino C57Bl/6) was injected with 500 B16 LeptoM cells intracisternally, as described in Remsik et al.^74^. Two weeks after the injection, the mouse was deeply anaesthetized and transcardially perfused with PBS (MSK Media Core). Tissues, including the brain, were dissected and immediately placed into a tube containing histology-grade PFA (4%; Sigma-Aldrich, HT501128). After overnight incubation, tissue was rinsed with water and submerged in 70% ethanol. The brain was cut coronally into four 2–3-mm-thick sections, placed into a tissue cartridge and embedded in formalin using routine, automated procedure. The embedded tissue was stored at room temperature.

For snRNA-seq-FFPE, a 100-μm-thick section of tissue was pre-processed on a prototype Singulator system. The sample was automatically processed in a NIC+ cartridge (S2 Genomics, 100-215-389) by three 10-min deparaffinization steps (CitriSolv, VWR), rehydrated by successive 1-ml washes of 100%, 100%, 70%, 50% and 30% ethanol and followed by two washes of PBS. The sample was then spun at 1,000*g* for 3 min and resuspended in 0.5 ml of Nuclei Isolation Reagent (NIR, S2 Genomics, 100-063-396) with 0.1 U ml^−1^ RNase inhibitor (Protector, MilliporeSigma, 3335399001); all subsequent solutions had RNase inhibitor. The sample was dissociated to single nuclei in a second NIC+ cartridge with 2 ml of NIR for 10 min, followed by a 2-ml wash with Nuclei Storage Reagent (NSR, S2 Genomics, 100-063-405). The single-nucleus suspension was spun at 500*g* for 5 min, resuspended in NSR and counted, and then snRNA-seq was performed on the Chromium instrument (10x Genomics) following the user guide manual for Chromium Fixed RNA Kit, Mouse Transcriptome (SinglePlex). Final libraries were sequenced on an Illumina NovaSeq S4 (R1: 28 cycles; i7: eight cycles; R2: 90 cycles).

To perform Xenium spatial profiling, FFPE mouse brain tissue adjacent to that used for snRNA-seq was sectioned into 5-μm-thick slices with a microtome and placed onto the sample area of a Xenium slide (10x Genomics). Profiling was conducted following the 10x Genomics User Guide (CG000578, CG000580 and CG000582). In brief, tissue slices were baked at 42 °C for 3 h and stored overnight in a desiccating chamber. The tissue was then deparaffinized, serially rehydrated and de-crosslinked, before overnight hybridization with gene-specific padlock probes (Mouse Brain Panel, 10x Genomics). After this, the probes were ligated and amplified to generate the rolling circle amplification (RCA) product, which was then prepared for imaging with the Xenium. Before imaging, tissue autofluorescence was suppressed, and DAPI was applied as counterstain. The Xenium was loaded with the necessary reagents for decoding the RCA products, in conjunction with the selection of regions of interest for imaging based on the DAPI images captured by the Xenium.

### Reporting summary

Further information on research design is available in the Nature Portfolio Reporting Summary linked to this article.

## Online content

Any methods, additional references, Nature Portfolio reporting summaries, source data, extended data, supplementary information, acknowledgements, peer review information; details of author contributions and competing interests; and statements of data and code availability are available at 10.1038/s41587-024-02193-4.

## Supplementary information

Reporting Summary

Supplementary Table All tables, each in a dedicated Excel sheet

## Data Availability

Raw sequencing data and processed count matrices for snRNA-seq from brain tissue bearing a leptomeningeal metastasis are publicly available in the Gene Expression Omnibus (https://www.ncbi.nlm.nih.gov/geo/) under accession number GSE246395. Segmented and processed Xenium data are publicly available through Zenodo (https://zenodo.org/) under accession number 10712720.

## Code availability

ENVI and COVET are available as Python packages at https://github.com/dpeerlab/ENVI and can be directly installed via ‘pip’ with the command ‘pip install scENVI’. A Jupyter notebook with an ENVI tutorial that reproduces motor cortex MERFISH results is available at github.com/dpeerlab/ENVI/blob/main/MOp_MERFISH_tutorial.ipynb.
