# Supplementary material for: The covariance environment defines cellular niches for spatial inference
Source: Nat Biotechnol. 2024 Apr 2;43(2):269–80. doi: 10.1038/s41587-024-02193-4 (PMC11445396; doi:10.1038/s41587-024-02193-4)
Supplement: Supplementary file 1 — Reporting Summary [file 41587_2024_2193_MOESM1_ESM.pdf]

nature portfolio

Corresponding author(s): Dana Pe'er

Last updated by author(s): Oct 30, 2023

Reporting Summary

Nature Portfolio wishes to improve the reproducibility of the work that we publish. This form provides structure for consistency and transparency in reporting. For further information on Nature Portfolio policies, see our Editorial Policies and the Editorial Policy Checklist.

Statistics

For all statistical analyses, confirm that the following items are present in the figure legend, table legend, main text, or Methods section.

n/a	Confirmed	
☐	☒	The exact sample size (*n*) for each experimental group/condition, given as a discrete number and unit of measurement
☐	☒	A statement on whether measurements were taken from distinct samples or whether the same sample was measured repeatedly
☐	☒	The statistical test(s) used AND whether they are one- or two-sided<br>*Only common tests should be described solely by name; describe more complex techniques in the Methods section.*
☐	☒	A description of all covariates tested
☐	☒	A description of any assumptions or corrections, such as tests of normality and adjustment for multiple comparisons
☐	☒	A full description of the statistical parameters including central tendency (e.g. means) or other basic estimates (e.g. regression coefficient) AND variation (e.g. standard deviation) or associated estimates of uncertainty (e.g. confidence intervals)
☐	☒	For null hypothesis testing, the test statistic (e.g. *F*, *t*, *r*) with confidence intervals, effect sizes, degrees of freedom and *P* value noted<br>*Give P values as exact values whenever suitable.*
☒	☐	For Bayesian analysis, information on the choice of priors and Markov chain Monte Carlo settings
☒	☐	For hierarchical and complex designs, identification of the appropriate level for tests and full reporting of outcomes
☐	☒	Estimates of effect sizes (e.g. Cohen's *d*, Pearson's *r*), indicating how they were calculated

Our web collection on statistics for biologists contains articles on many of the points above.

Software and code

Policy information about availability of computer code

Data collection	cellranger-7.1.0, 10x Xenium processing pipeline (https://www.10xgenomics.com/support/in-situ-gene-expression/documentation/steps/onboard-analysis/xenium-algorithms-overview)
Data analysis	python 3.8, tensorflow 2.8.4, scanpy 1.9.3, sklearn 1.2.2, scipy 1.10.1, pandas 1.5.3, numpy 1.22.4, umap 0.5.3, phenograph 1.5.7

For manuscripts utilizing custom algorithms or software that are central to the research but not yet described in published literature, software must be made available to editors and reviewers. We strongly encourage code deposition in a community repository (e.g. GitHub). See the Nature Portfolio guidelines for submitting code & software for further information.

Data

Policy information about availability of data

All manuscripts must include a data availability statement. This statement should provide the following information, where applicable:

- Accession codes, unique identifiers, or web links for publicly available datasets
- A description of any restrictions on data availability
- For clinical datasets or third party data, please ensure that the statement adheres to our policy

Raw sequencing data and processed count matrices for snRNA-seq from brain tissue bearing a leptomeningeal metastasis is publicly available in GEO ((https://www.ncbi.nlm.nih.gov/geo/) under accession GSE246395. Segmented and processed Xenium data is publicly available through Zenodo (https://zenodo.org/) under

accession 10712720.

Human research participants

Policy information about studies involving human research participants and Sex and Gender in Research.

Reporting on sex and gender	No human data is included in our study
Population characteristics	*Describe the covariate-relevant population characteristics of the human research participants (e.g. age, genotypic information, past and current diagnosis and treatment categories). If you filled out the behavioural & social sciences study design questions and have nothing to add here, write "See above."*
Recruitment	*Describe how participants were recruited. Outline any potential self-selection bias or other biases that may be present and how these are likely to impact results.*
Ethics oversight	*Identify the organization(s) that approved the study protocol.*

Note that full information on the approval of the study protocol must also be provided in the manuscript.

Field-specific reporting

Please select the one below that is the best fit for your research. If you are not sure, read the appropriate sections before making your selection.

☒ Life sciences ☐ Behavioural & social sciences ☐ Ecological, evolutionary & environmental sciences

For a reference copy of the document with all sections, see nature.com/documents/nr-reporting-summary-flat.pdf

Life sciences study design

All studies must disclose on these points even when the disclosure is negative.

Sample size	Sample sizes (cell numbers) for snRNA-seq and Xenium datasets collected by us were determined based on filtering and processing by CellRanger and the Xenium pipeline from 10x Genomics. All other data in the manuscript was published by other groups and study designs are described in their respective publications.
Data exclusions	Data was not excluded. Only individual cells from single-cell and spatial samples were removed based on quality and library size. Full filtering details are available in the Methods.
Replication	ENVI and COVET were shown to be robust to data sparsity and neighborhood size based on replicated in silico ablation studies. In addition, we demonstrated consistency between ENVI imputation of missing genes onto spatial data, and ENVI inference of spatial context onto dissociated single-cell data.
Randomization	For benchmarking the gene imputation performance of different algorithms on spatial datasets with hundreds of genes, we performed k-fold (k = 5) cross validation. For each relevant dataset, genes were randomly divided into 5 groups of approximately equal size. Every algorithm was trained to reconstruct the expression of one group based on the remaining 4 groups, and accuracy was measured between reconstructed and true expression profiles.
Blinding	No blinding was nescessary as our study is exploratory.

Reporting for specific materials, systems and methods

We require information from authors about some types of materials, experimental systems and methods used in many studies. Here, indicate whether each material, system or method listed is relevant to your study. If you are not sure if a list item applies to your research, read the appropriate section before selecting a response.

Materials & experimental systems

n/a	Involved in the study	
☒	☐	Antibodies
☒	☐	Eukaryotic cell lines
☒	☐	Palaeontology and archaeology
☐	☒	Animals and other organisms
☒	☐	Clinical data
☒	☐	Dual use research of concern

Methods

n/a	Involved in the study	
☒	☐	ChIP-seq
☒	☐	Flow cytometry
☒	☐	MRI-based neuroimaging

Animals and other research organisms

Policy information about studies involving animals; ARRIVE guidelines recommended for reporting animal research, and Sex and Gender in Research

Laboratory animals	A single 8-week-old female C57Bl/6-Tyrc-2 (JAX #000058, albino C57Bl/6) was used in this study.
Wild animals	Wild animals were not used in the study.
Reporting on sex	Xenium profiling was conducted on brain isolated from a single female mouse bearing melanoma leptomeningeal metastasis. Sex was not considered in the study design.
Field-collected samples	No field samples were collected.
Ethics oversight	Animal study was approved by the MSK Institutional Animal Care and Use Committee, under the protocol 18-01-002.

Note that full information on the approval of the study protocol must also be provided in the manuscript.
